# Association of estimated plasma volume status with progression from stage 2 to stage 4 cardiovascular–kidney–metabolic syndrome: a cohort from the Tianjin region of China

**DOI:** 10.3389/fendo.2026.1959815

**Published:** 2026-10-15

**Authors:** Longrong Huang, Xiaofei Luo, Xiangqin Ou, Yu Liu, Lin Zhang, Qiang Xu

**Affiliations:** 1 Department of Cardiology, Second Affiliated Hospital of Tianjin University of Traditional Chinese Medicine, Tianjin, China; 2 First Teaching Hospital of Tianjin University of Traditional Chinese Medicine, National Clinical Research Center for Chinese Medicine Acupuncture and Moxibustion, Tianjin, China; 3 State Key Laboratory of Component-based Chinese Medicine, Tianjin University of Traditional Chinese Medicine, Tianjin, China

**Keywords:** cardiovascular–kidney–metabolic syndrome, cohort study, estimated plasma volume status, risk stratification, volume management

## Abstract

**Introduction:**

Cardiovascular-kidney-metabolic syndrome (CKM) is a systemicdisorder linked to insulin resistance. The association between estimated plasmavolume status (ePVS) and progression from CKM stage 2 (CKM2) to stage 4(CKM4) remains poorly understood. This study assessed the association betweenePVS and CKM2-to-CKM4 progression.

**Methods:**

A total of 36,505 hospitalized CKM2 patients from the TJHFIT cohortwere followed for 10 years, with 15,495 incident CKM4 events. Cox proportionalhazards models, restricted cubic splines, and SHAP analyses were applied toestimate hazard ratios, nonlinear associations, and variable importance, adjustedfor demographics, medical history, laboratory parameters, and medications.

**Results:**

CKM4 patients had significantly higher admission and discharge ePVS thancontrols (all P < 0.05). In quartile-stratified analyses, elevated risk of CKM4progression was independently observed only in the upper admission ePVS strata(Q3 and Q4), with no significant risk in the moderate stratum (Q2). For dischargeePVS, a significant risk signal emerged starting from Q2. The highest admission ePVSquartile was associated with 43%, 88%, and 76% higher risks of CKM4, CKM4b, andheart failure versus Q1. Restricted cubic spline analysis revealed a spoon-shapednonlinear association between admission ePVS and CKM4, with an inflection point atapproximately 5.14. The association was stronger in patients aged >60 years.

**Discussion:**

Higher admission ePVS (Q3/Q4) is independently associated with CKM2-to-CKM4 progression and may serve as a noninvasive risk biomarker particularly in older high-risk populations. External cohort validation is requiredbefore clinical application.

**Trial registration:**

The research has been registered (ChiCTR2300077220) with ethics approval (TYLL2023-K-016).

## Graphical Abstract

**Figure:**
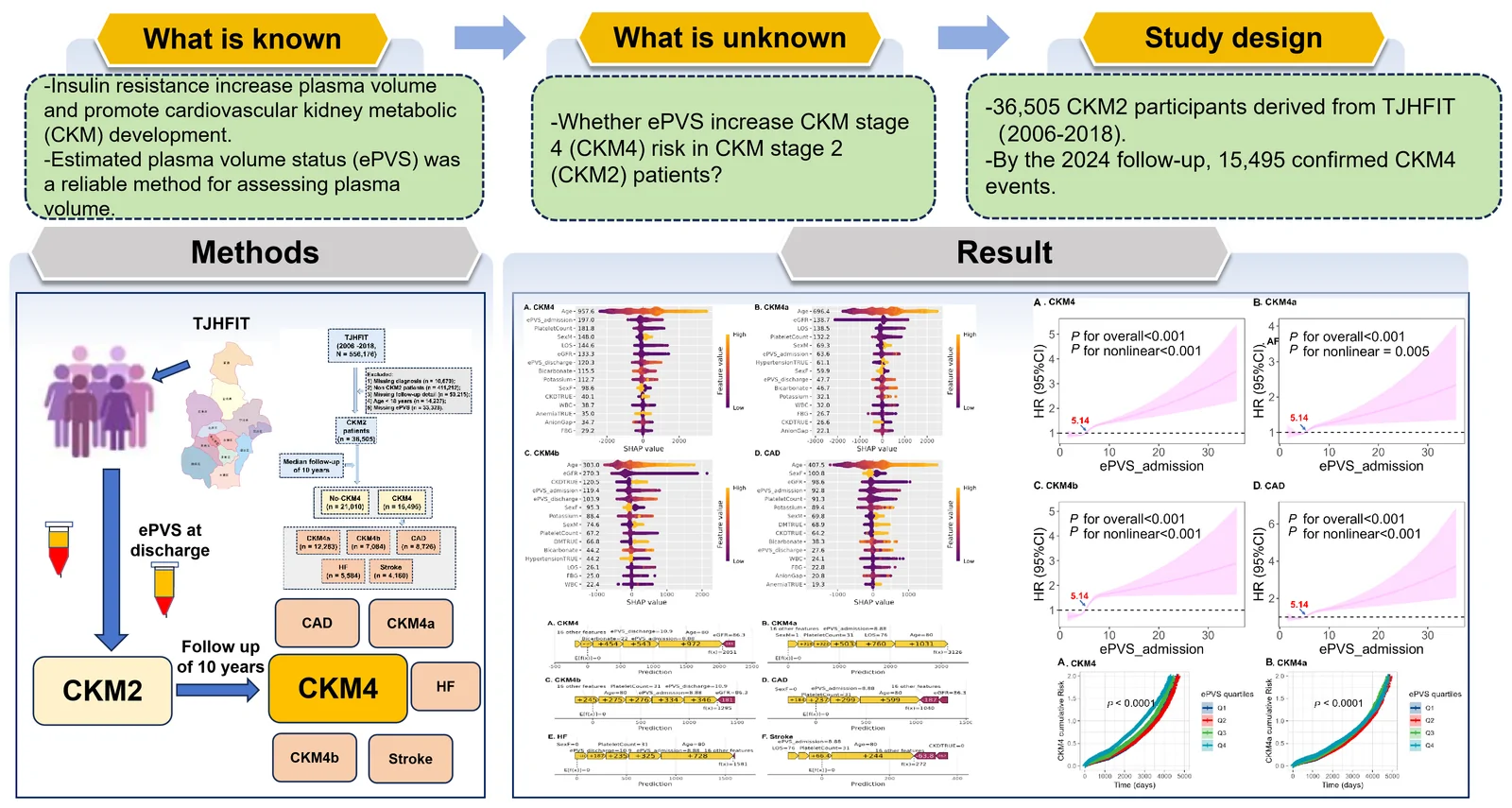

## 1 Introduction

Cardiovascular–kidney–metabolic (1) syndrome (CKM) represents a complex pathophysiological state characterized by the interplay between cardiovascular diseases, chronic kidney disease, and metabolic disorders. While the relationship between CKM and insulin resistance (2) has been extensively studied, the connection between CKM and plasma volume status remains underexplored. First, insulin resistance has been shown to increase plasma volume (3). Second, the change in volume status can influence both renal (4) and cardiovascular prognoses (5). Therefore, we hypothesize that the plasma volume may play a significant role in the progression of CKM.

The gold-standard methods for assessing plasma volume have traditionally relied on invasive and complex techniques, such as hemodynamic measurements or the use of radioisotope tracers (6, 7). These methods are not suitable for routine clinical practice because of their technical demands and associated risks. Over the past decade, noninvasive approaches have led to the development of the estimation of plasma volume status (ePVS) (8), a simpler and more accessible method that has proven to be both accurate and reliable. While ePVS (9) has demonstrated strong predictive value for various cardiovascular (10) and metabolic diseases (11, 12), the correlation between ePVS and CKM remains unclear.

Although CKM is classified into stages 0 to 4 (13), the 0th and 1st stages are typically characterized by a clinically asymptomatic phase, where pathological changes are not yet significant. At these stages, changes in plasma volume may be subtle and have not yet caused a substantial association with cardiovascular and renal function. Consequently, observing significant changes in plasma volume is challenging when studying the transitional phase from CKM0 or CKM1. In contrast, from CKM2 to CKM4, both pathological and physiological changes become more pronounced. CKM2 (1) is often associated with metabolic abnormalities, such as insulin resistance and mild renal dysfunction. Therefore, studying the progression from CKM2 to CKM4 offers a clearer understanding of the critical role that plasma volume plays in CKM.

In summary, the primary aim of this study was to investigate the relationship between ePVS and the progression of CKM from CKM2 to CKM4. Additionally, this study evaluated the potential of ePVS as a predictive tool for identifying patients at risk of transitioning from CKM2 to CKM4. The findings from this research could provide valuable insights into the pathophysiology of CKM and offer a novel, noninvasive approach for monitoring disease progression.

## 2 Methods

### 2.1 Study participants

This study included the Tianjin Heart Failure with Integrated Treatment (TJHFIT) cohort (11), which was registered (ChiCTR2300077220), to explore the therapeutic effects of traditional Chinese and Western medicine in treating heart failure with a preserved ejection fraction in the Tianjin region. The participants were enrolled from 2006 to 2018 from 43 hospitals in Tianjin, China.

The hospitalized study population was selected on the basis of the following exclusion criteria: (1) missing baseline diagnosis data, (2) non-CKM2 patients—this step excluded individuals in CKM stages 0 and 1 as well as those with prevalent subclinical or clinical cardiovascular disease (CVD) at baseline (CKM stages 3 and 4, including coronary artery disease, atrial fibrillation, and heart failure), (3) missing complete follow-up details, (4) age <18 years, and (5) missing estimated plasma volume status (ePVS) measurements at either hospital admission or discharge. Consequently, the final enrolled cohort consisted exclusively of patients with CKM stage 2.

According to the 2023 American Heart Association (AHA) Presidential Advisory on Cardiovascular–Kidney–Metabolic Health and Disease, CKM stage 2 is characterized by the presence of metabolic risk factors (including hypertriglyceridemia, hypertension, diabetes mellitus, or metabolic syndrome) or moderate- to high-risk chronic kidney disease (CKD) in the absence of clinical cardiovascular disease. In our study, participants were considered eligible for CKM stage 2 if they fulfilled at least one of these criteria. Individuals with isolated excess adiposity or prediabetes without additional metabolic risk factors or CKD were excluded.

As previous studies have indicated (10), ePVS is a verified method to test the plasma volume, and following is the calculation method of ePVS (mL/g) = $\frac{100-\text{Hematocrit }(\text{HCT},\text{ \%})}{\text{Hemoglobin }(\text{Hb},\text{ g}/\text{dL})}$. In this calculation, hematocrit is input as a percentage value (e.g., 40 for 40% hematocrit) rather than a decimal fraction. When Hct is expressed as a percentage (representing the milliliters of red blood cells per 100 mL of whole blood) and Hb is expressed in grams per deciliter (g/dL), the unit of the resulting ePVS is dimensionally consistent as milliliters per gram (mL/g), reflecting plasma volume relative to hemoglobin mass.

For illustrative purposes, in an individual with 40% Hct and 14 g/dL Hb, ePVS is calculated as (100 − 40)/14 ≈ 4.29 mL/g.

To assess whether the predictive value of the ePVS is stable, we also included the ePVS at admission and discharge during hospitalization. Finally, a total of 36,505 CKM2 patients were included (**Figure 1**).

**Figure 1:**
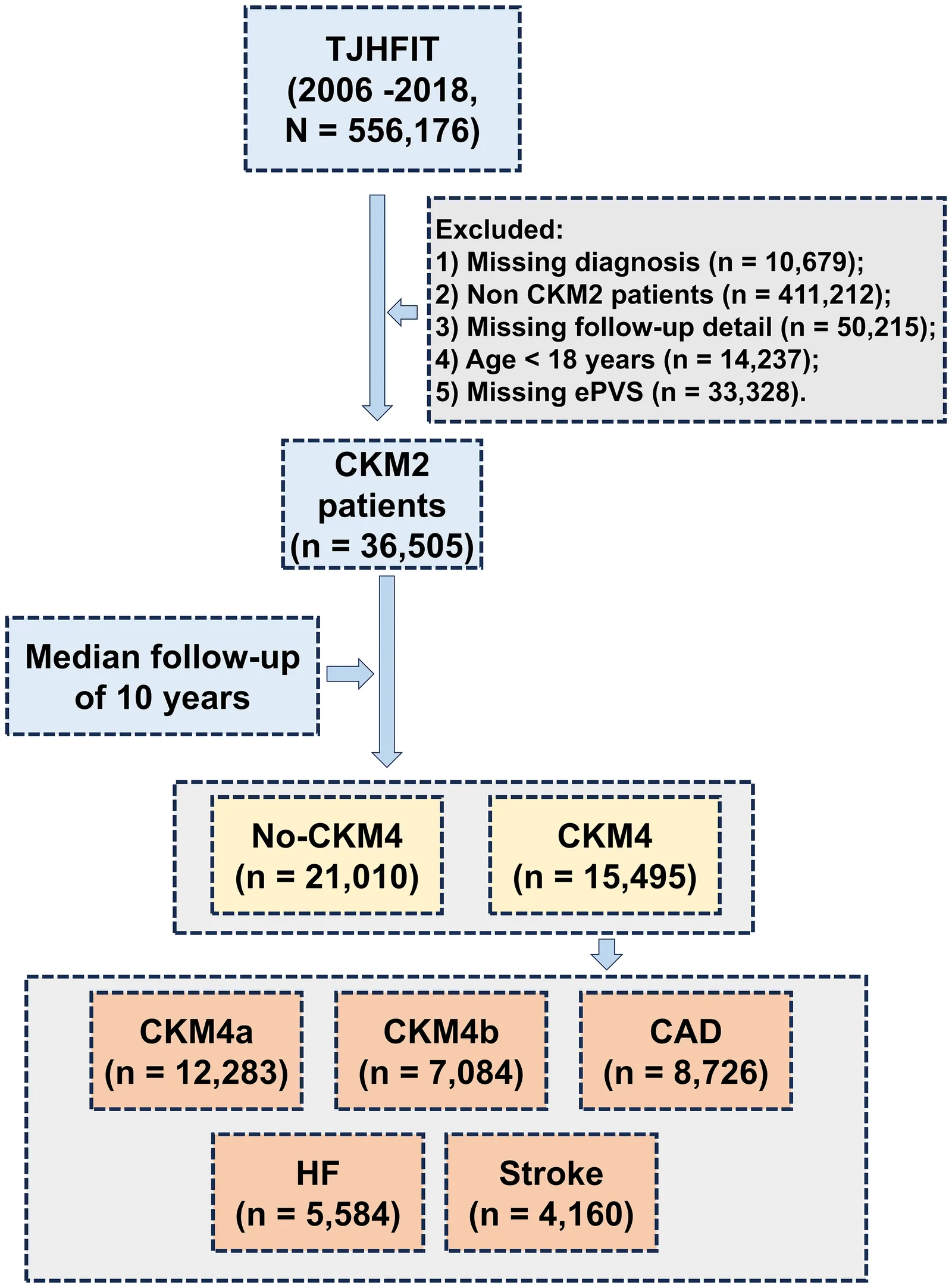
Flow chart of the study (because a CKM2 patient may experience multiple endpoint diseases simultaneously, the total number of patients with CKM4a, CKM4b, CAD, HF, and stroke is greater than that of CKM4 patients).

### 2.2 Definition of the endpoint

In accordance with the 2023 AHA CKM staging framework (13), CKM stage 4 was explicitly defined as an incident outcome in our analysis. Specifically, the endpoint was the new occurrence of clinical cardiovascular disease (CVD) during the follow-up period. We defined CVD as CAD, stroke, peripheral arterial disease, heart disease caused by various factors, deep vein thrombosis, or pulmonary embolism. Whenever the abovementioned disease occurred, it was defined as an incident CVD event. The CKM4 patients were classified as CKM4a (without kidney failure) or CKM4b (with kidney failure) according to whether they presented with kidney failure. More importantly, CKM4-related diseases, including CAD, HF, and stroke, were further explored. CAD was defined as myocardial infarction, coronary artery disease, angina, ischemic cardiomyopathy, valvular heart disease, mitral valve insufficiency, aortic valve stenosis, cardiomyopathy, aortocoronary bypass graft, or prosthetic heart valve. HF was defined as heart failure, left ventricular failure, or cardiogenic shock. The strokes included various strokes, cerebral infarction, cerebral ischemia, subarachnoid hemorrhage, intracerebral hemorrhage, transient ischemic attack, and sequelae of cerebrovascular disease. Kidney failure was defined as CKD patients with an estimated glomerular filtration rate (eGFR) <15 mL/(min·1.73 m^2^), end-stage renal disease, dialysis, or kidney transplant. The follow-up period ended when the patient developed CKM4 or by April 2024, and the median follow-up time was 10 years. Because all participants with prevalent clinical CVD at baseline were excluded according to our study design, all CKM4 events captured during follow-up represented new-onset clinical CVD occurring after CKM stage 2 enrollment.

### 2.3 Statistical analyses

All of the statistical computations were performed via R statistical software (v4.4.1). Continuous variables were evaluated for a Gaussian distribution through both formal testing (Shapiro–Wilk) and graphical methods. On the basis of distributional characteristics, between-group comparisons employed either independent samples *t*-tests (parametric) or Mann–Whitney *U*-tests (nonparametric). For categorical data analyses, Pearson’s chi-square tests were used. CKM2 patients whose sex, age, medical history, and medication history were missing were excluded. In the laboratory results, variables with more than 5% missing values were also excluded. Variables with less than 5% missing values were imputed via the *mice* package for multiple imputation. Multiple imputation was performed using the mice package in R (version 4.4.1). Continuous variables were imputed using predictive mean matching (PMM), and binary variables were imputed using logistic regression imputation (logreg), which are the default methods in mice for numeric and two-level factor variables, respectively. Five imputed datasets (*m* = 5) were generated with 10 iterations per imputation (maxit = 10). Pooling of estimates across imputed datasets was performed according to Rubin’s rules, implemented via the pool() function in the mice package. Missing data were assumed to be missing at random (MAR), which is reasonable for observational cohort studies. With all missing rates below 5%, bias is likely limited, though residual influence cannot be fully excluded.

Multicollinearity among the variables included in model 4 was assessed using the variance inflation factor (VIF) to ensure the stability of the regression estimates. To further assess the robustness of the fully adjusted model, a VIF analysis was performed for all included covariates. All VIF values were well below the commonly used threshold of 5, with the maximum value being 1.75 for hypertension. This indicates that multicollinearity is not a substantial problem in our model (**Supplementary Table S1**). To address the potential bias from competing risks, we additionally performed Fine–Gray subdistribution hazard models, with all-cause mortality during follow-up treated as the primary competing event for the endpoint of CKM4b progression.

Shapley Additive exPlanations (SHAP) analysis was performed using the treeshap R package to assess the contribution of individual predictors to the predictions generated by endpoint-specific random survival forest models. Separate random survival forest models were fitted for each of the six endpoints. The hyperparameters were set as follows: number of trees = 1,000, maximum depth = 10, mtry = 5, minimum node size = 3, splitrule = “logrank”, sample.fraction = 1, and replace = TRUE. A fixed random seed was used to ensure reproducibility, and the models were run in single-thread mode. For each endpoint, SHAP values were calculated from the predictions generated by the corresponding random survival forest model to quantify the contribution of each predictor to the model prediction. Model performance was evaluated using out-of-bag (OOB) predictions, with discrimination assessed by the C-index and prediction error assessed by Brier scores at 1, 3, and 5 years.

A Cox proportional hazards model was used to calculate the hazard ratio (HR) and 95% confidence intervals (CIs) of ePVSs for CKM4. Five Cox models were used: model 0 was unadjusted; model 1 was adjusted for age, sex, and length of stay; model 2 was adjusted for model 1 and medical history; model 3 was adjusted for model 2 and laboratory results; and model 4 was adjusted for model 3 and medication history. To account for potential competing risks, Fine–Gray subdistribution hazard models were additionally performed for the progression to CKM4b, treating all-cause mortality and CKM4a as competing events, respectively. The Fine–Gray models were adjusted for the same covariates as model 4 in the primary Cox regression analyses.

Importantly, to optimize the trend for ePVS in CKM4, a restricted cubic spline (RCS) (14) was applied. The RCS model (with four knots) was used to examine the optimal ePVS at various endpoints. To better explore the trend for ePVSs, we quartile the ePVSs (both admission and discharge in the hospital).

## 3 Results

### 3.1 General characteristics

A total of 36,505 hospitalized CKM2 patients were included, covering 16,388 (45%) men with a median age of 61 years, and 15,495 ultimately developed CKM4 (**Table 1**). Patients with CKM2 have hypertension as the primary condition, followed by CKD, DM, and anemia. The overall medical history revealed 11,780 (32%) patients with DM, 25,391 (70%) with hypertension, 1,670 (4.6%) with hypertriglyceridemia, 65 (0.2%) with MetS, 11,926 (33%) with CKD, 9,169 (25%) with anemia, 4,128 (11%) with hypothyroidism, and 1,475 (4.0%) with hyperuricemia.

**Table 1:** Baseline characteristics of CKM2 patients who progress to CKM4 or non-CKM.

Characteristics	Overall	Non-CKM4	CKM4	*P*-value
	*N* = 36,505	*N* = 21,010	*N* = 15,495	
Demographic				
Sex (male), *n* (%)	16,388 (45%)	8,977 (43%)	7,411 (48%)	<0.001
Age, years	61 (50, 72)	58 (45, 69)	66 (56, 76)	<0.001
LOS, days	3.0 (1.0, 5.0)	3.0 (1.0, 6.0)	3.0 (1.0, 5.0)	<0.001
Medical history				
DM, *n* (%)	11,780 (32%)	6,159 (29%)	5,621 (36%)	<0.001
Hypertension, *n* (%)	25,391 (70%)	14,718 (70%)	10,673 (69%)	0.016
Hypertriglyceridemia, *n* (%)	1,670 (4.6%)	1,039 (4.9%)	631 (4.1%)	<0.001
MetS, *n* (%)	65 (0.2%)	50 (0.2%)	15 (<0.1%)	0.002
CKD, *n* (%)	11,926 (33%)	5,798 (28%)	6,128 (40%)	<0.001
Anemia, *n* (%)	9,169 (25%)	5,162 (25%)	4,007 (26%)	0.005
Hypothyroidism, *n* (%)	4,128 (11%)	2,311 (11%)	1,817 (12%)	0.03
Hyperuricemia, *n* (%)	1,475 (4.0%)	699 (3.3%)	776 (5.0%)	<0.001
Medication history				
Metformin, *n* (%)	2,297 (6.3%)	1,211 (5.8%)	1,086 (7.0%)	<0.001
Glimepiride, *n* (%)	71 (0.2%)	31 (0.1%)	40 (0.3%)	0.018
Furosemide, *n* (%)	4,963 (14%)	2,378 (11%)	2,585 (17%)	<0.001
Beta-block, *n* (%)	6,862 (19%)	2,875 (14%)	3,987 (26%)	<0.001
Statin, *n* (%)	5,654 (15%)	2,579 (12%)	3,075 (20%)	<0.001
CCB, *n* (%)	6,724 (18%)	3,277 (16%)	3,447 (22%)	<0.001
ARB, *n* (%)	3,276 (9.0%)	1,544 (7.3%)	1,732 (11%)	<0.001
ACEI, *n* (%)	8,918 (24%)	4,420 (21%)	4,498 (29%)	<0.001
Laboratory results				
ePVS_admission, (mL/g), median (IQR)	5.14 (4.39, 6.18)	5.09 (4.36, 6.11)	5.20 (4.43, 6.28)	<0.001
ePVS_discharge, (mL/g), median (IQR)	5.77 (4.82, 7.11)	5.72 (4.79, 7.07)	5.83 (4.86, 7.16)	<0.001
eGFR, mL/(min·1.73 m^2^), median (IQR)	75 (54, 97)	80 (58, 103)	68 (49, 89)	<0.001
Anion gap, mEq/L, median (IQR)	15.0 (13.0, 17.0)	14.0 (12.0, 17.0)	15.0 (13.0, 17.0)	<0.001
Bicarbonate, mEq/L, median (IQR)	25.0 (23.0, 27.0)	25.0 (22.0, 27.0)	25.0 (23.0, 28.0)	<0.001
Chloride, mEq/L, median (IQR)	102.0 (99.0, 105.0)	102.0 (99.0, 105.0)	102.0 (99.0, 105.0)	0.2
Creatinine, mg/dL, median (IQR)	0.90 (0.70, 1.20)	0.90 (0.70, 1.20)	1.00 (0.80, 1.40)	<0.001
FBG, mg/dL, median (IQR)	116 (98, 148)	114 (98, 145)	117 (99, 153)	<0.001
Hematocrit, %, median (IQR)	36.9 (32.9, 40.4)	37.0 (33.1, 40.6)	36.6 (32.6, 40.2)	<0.001
Hemoglobin, g/dL, median (IQR)	12.30 (10.80, 13.60)	12.40 (10.90, 13.60)	12.20 (10.70, 13.50)	<0.001
MCHC, g/dL, median (IQR)	33.30 (32.40, 34.30)	33.40 (32.40, 34.30)	33.30 (32.30, 34.30)	<0.001
MCH, pg, median (IQR)	30.00 (28.50, 31.50)	30.00 (28.50, 31.50)	30.00 (28.50, 31.50)	0.7
MCV, fL, median (IQR)	90 (86, 94)	90 (86, 94)	90 (86, 94)	0.001
Platelet count, K/uL, Median (IQR)	237 (183, 301)	239 (185, 302)	234 (180, 299)	<0.001
Potassium, mEq/L, median (IQR)	4.10 (3.80, 4.50)	4.10 (3.80, 4.50)	4.20 (3.80, 4.60)	<0.001
RDW, %, median (IQR)	13.80 (13.10, 14.90)	13.70 (13.00, 14.70)	13.90 (13.20, 15.10)	<0.001
RBC, m/uL, median (IQR)	4.13 (3.65, 4.56)	4.15 (3.68, 4.58)	4.09 (3.60, 4.53)	<0.001
Sodium, mEq/L, median (IQR)	138.0 (136.0, 141.0)	138.0 (136.0, 141.0)	139.0 (136.0, 141.0)	0.11
WBC, K/uL, median (IQR)	8.6 (6.5, 11.4)	8.8 (6.6, 11.7)	8.3 (6.4, 11.1)	<0.001

Compared with non-CKM4 patients, CKM4 patients (**Table 1**) had significantly greater ePVSs, with medians (IQRs) of 5.20 (4.43, 6.28) vs. 5.09 (4.36, 6.11) at admission and 5.83 (4.86, 7.16) vs. 5.72 (4.79, 7.07) at discharge, respectively. In terms of demographic and medical history, compared with non-CKM4 patients, CKM4 patients were older and were more likely to be male and have DM, CKD, anemia, hypothyroidism, and hyperuricemia. The laboratory results revealed that CKM4 patients had greater anion gaps, bicarbonate, creatinine, length of stay (LOS), fasting blood glucose (FBG), potassium, red blood cell distribution width (RDW), and sodium. To address the potential influence of missing data, we performed sensitivity analyses on the multiple imputation process. The missing data proportions for all variables were low, with the highest being 4.77% for platelet count and 4.76% for WBC (**Supplementary Table S2**).

Furthermore, the baseline characteristics of CKM2 patients who progressed to CKM4a, CKM4b, CAD, HF, or stroke were recorded (**Supplementary Tables S3**-**S7**). The stroke patients (**Supplementary Table S7**) had a significantly greater ePVS_discharge than the nonstroke patients did; the median (IQR) was 5.77 (4.82, 7.13) vs. 5.75 (4.81, 6.98), but ePVS_admission was not significant. Similar to CKM4 patients, CKM4a, CKM4b, CAD, and HF patients (**Supplementary Tables S3**-**S6**) had significantly greater ePVSs (both at admission and discharge) than non-CKM4a, non-CKM4b, non-CAD, and non-HF patients did.

### 3.2 SHAP for important variables

To observe the impact of variables on the endpoints, we used SHAP to assess the importance of the variables. The medication history, hemoglobin, hematocrit, and red-blood-cell-related indicators were deleted to avoid data overfitting (ePVS were calculated by hemoglobin and hematocrit; medical history was related to medication history). Finally, 21 variables were included, namely: sex, age, LOS, DM, hypertension, hypertriglyceridemia, MetS, CKD, anemia, hyperuricemia, anion gap, bicarbonate, FBG, chloride, platelet count, creatinine, potassium, WBC, ePVS_admission, ePVS_discharge, and eGFR.

For the abovementioned 21 variables, the SHAP-based random forest was used, and the top 15 significant variables for various endpoints are shown (**Figure 2**). In CKM4, the SHAP values of ePVS_admission and ePVS_discharge were 197 and 120, respectively, and ePVS_admission was less than age (957.6) but greater than any other variable. ePVS_admission had a higher SHAP value than ePVS_discharge did at each endpoint (**Figures 2A–F**), which indicated that ePVS_admission played a greater model-internal predictive contribution than ePVS_discharge did. Among all outcomes (**Figure 2**), the SHAP value of ePVS_admission was better than that of WBC and FBG (CKM poor prognosis indicators). In CKM4 (**Figure 2A**), HF (**Figure 2E**), and stroke (**Figure 2F**), ePVS_admission had a higher SHAP value than did eGFR, which indicated a more important role (**Supplementary Table S8**). The OOB C-index ranged from 0.6021 to 0.7287 across the six endpoints (**Supplementary Table S9**). SHAP values were used to rank the relative contribution of each variable within the survival random forest model. These values reflect model-internal predictive importance and should not be interpreted as direct clinical causal evidence.

**Figure 2:**
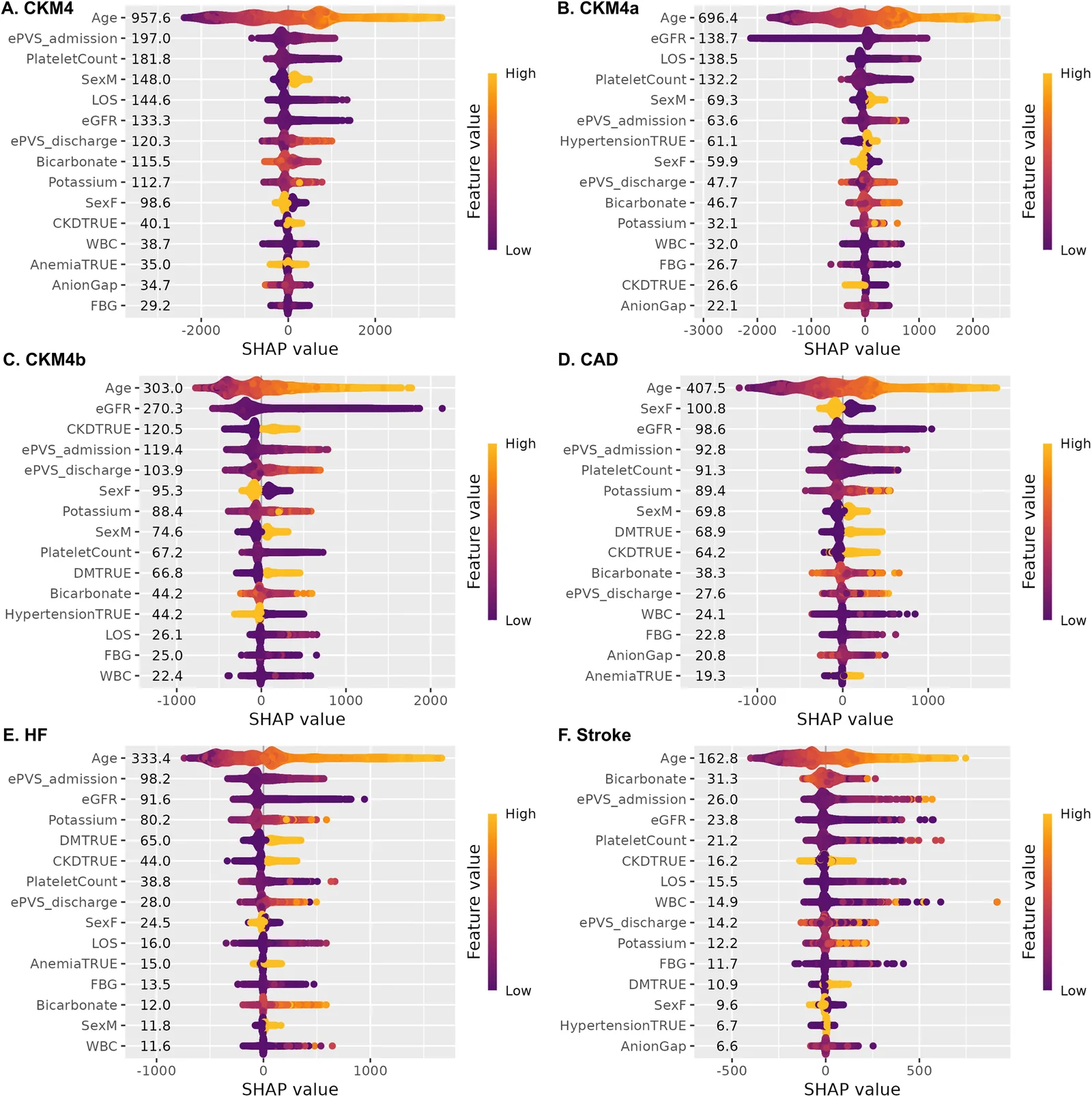
Top 15 important variables in SHAP analysis for various endpoints. **(A)** CKM4 progression; **(B)** CKM4a progression; **(C)** CKM4b progression; **(D)** coronary artery disease (CAD); **(E)** heart failure (HF); **(F)** stroke. The x-axis represents the SHAP value, which reflects the direction and magnitude of each feature’s contribution to model prediction. The color gradient from purple to orange indicates feature values ranging from low to high. Features are ranked by the average absolute SHAP value in descending order.SexF, female; SexM, male; LOS, length of stay; WBC, white blood cell count; FBG,fast blood glucose; DM, diabetes mellitus; eGFR, estimated glomerular filtration rate; CKD, chronic kidney disease. SHAP summary plots of feature importance for six clinical endpoints.

The force plots of various endpoints in SHAP were subsequently processed (**Figure 3**). In CKM4 (**Figure 3A**), age, bicarbonate, ePVS_admission, and ePVS_discharge were the top predictive features of the outcome. eGFR showed a negative SHAP contribution to the illustrated prediction. ePVS_admission showed a positive contribution to the predicted risk score in the illustrated cases (**Figures 3A–F**).

**Figure 3:**
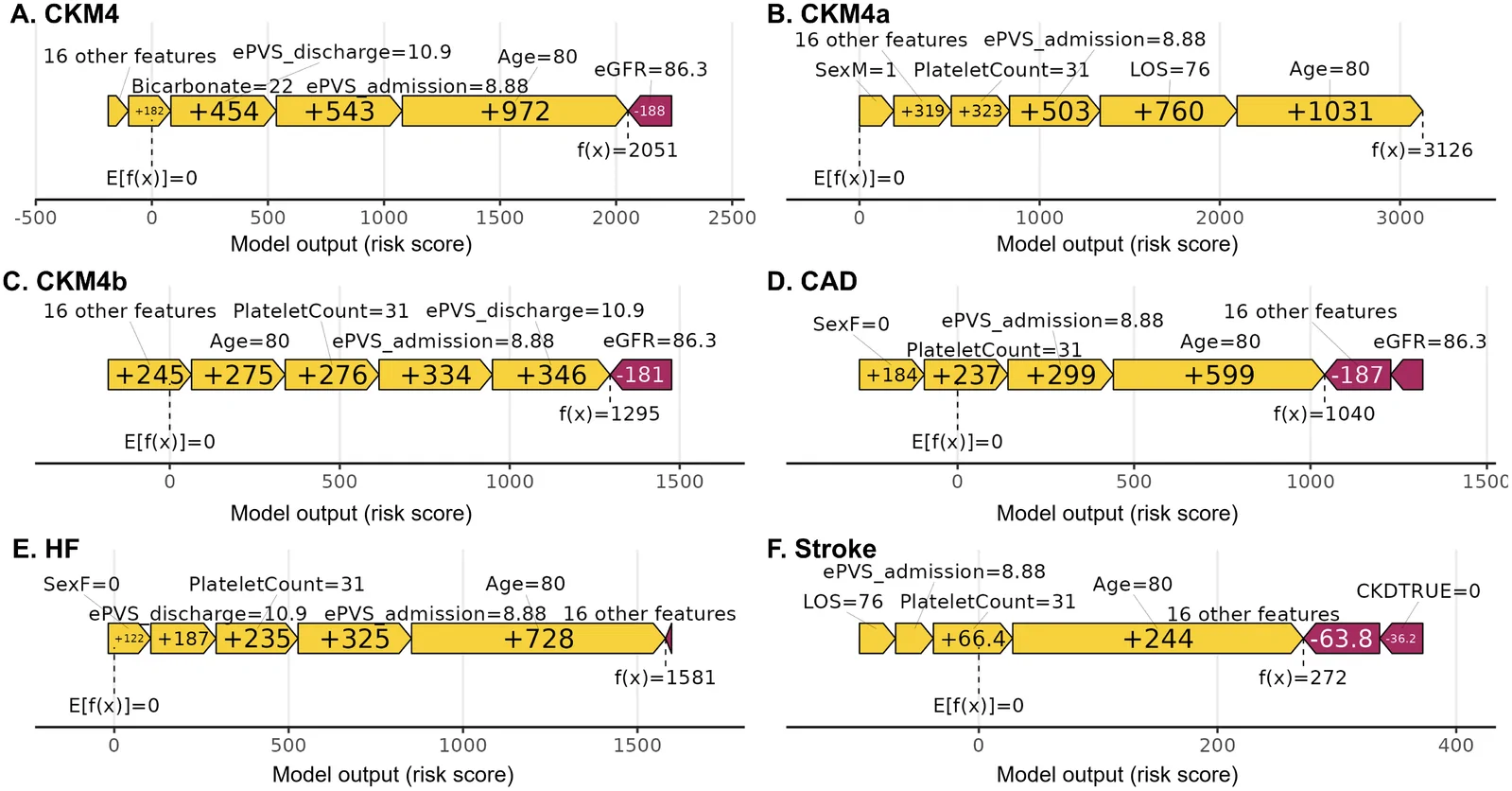
Force plots in SHAP analysis for various endpoints. **A)** CKM4 progression; **(B)** CKM4a progression; **(C)** CKM4b progression; **(D)** coronary artery disease (CAD); **(E)** heart failure (HF); **(F)** stroke. The xaxis represents the model output (risk score). Each colored segment represents the SHAP contribution of an individual feature to the modelprediction, with the numerical value within the segment indicating the magnitude of the corresponding SHAP value. Yellow segments indicate positive contributions that increase the predicted risk score, whereas magenta segments indicate negative contributions that decrease the predicted risk score.SexF, female; SexM, male; eGFR, estimated glomerular filtration rate; LOS, length of stay.

### 3.3 Cox analysis for ePVSs and endpoints

The Cox proportional hazards model for ePVS_admission (**Figure 4**) and ePVS_discharge (**Supplementary Figure S1**) is shown. For each unit of ePVS_admission in model 4, the risk of CKM2 patients progressing to CKM4, CKM4a, CKM4b, CAD, HF, and stroke increased by 7%, 5%, 9%, 7%, 10%, and 7%, respectively. For each unit of ePVS_discharge (**Supplementary Figure S1**) of model 4, the risk of CKM2 patients progressing to CKM4, CKM4a, CKM4b, CAD, HF, and stroke increased by 8%, 4%, 9%, 6%, 11%, and 5%, respectively. The HRs and 95% CIs were all significant in each model of the abovementioned endpoints, which indicated a stable predictive value for both ePVS_admission and ePVS_discharge.

**Figure 4:**
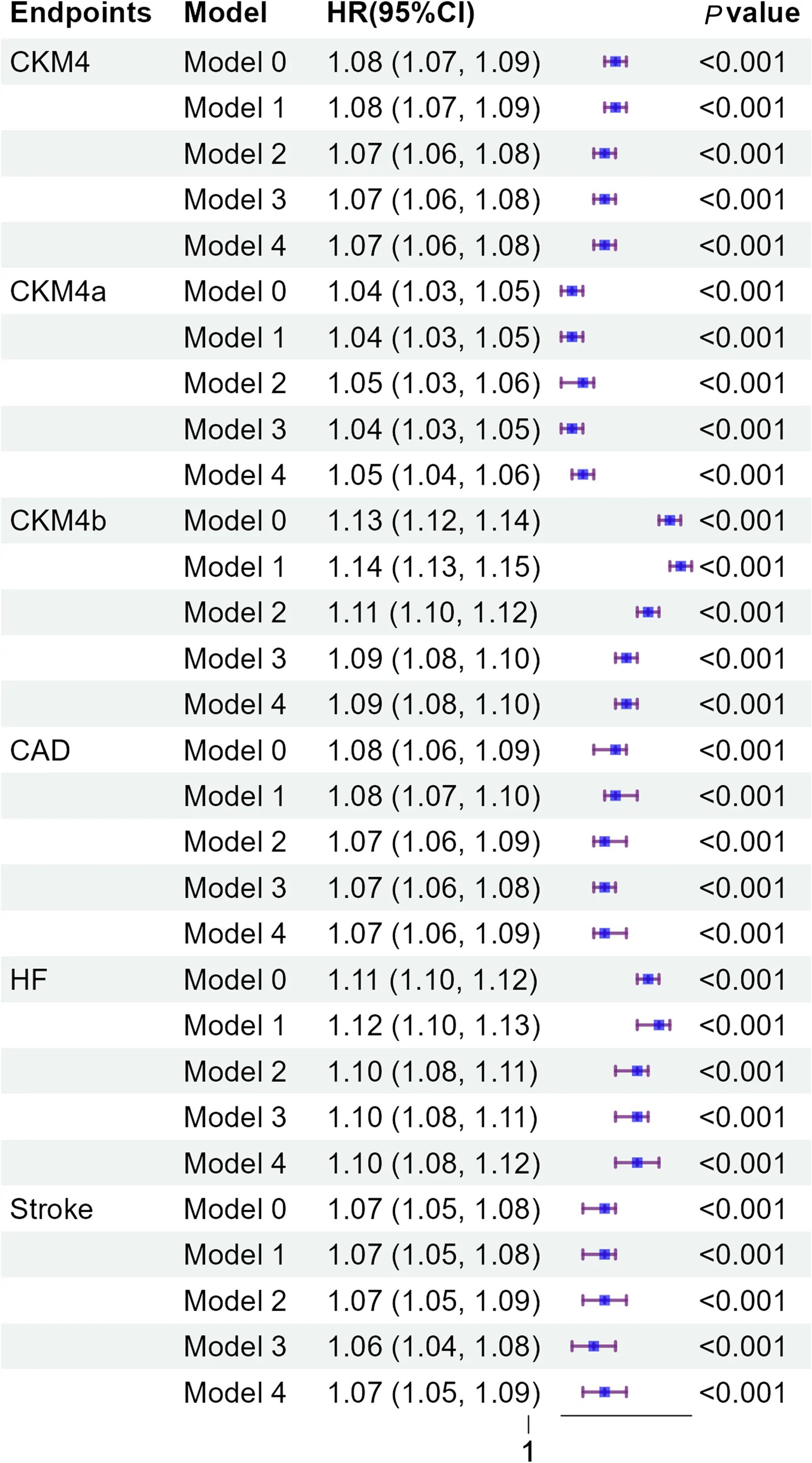
Forest plot for ePVS_admission with the five models. Model 0, unadjusted; model 1, adjusted for sex, age, and LOS; model 2, adjusted for model 1 and DM, hypertension, hypertriglyceridemia, MetS, CKD, anemia, and hyperuricemia; model 3, adjusted for model 2 and bicarbonate, chloride, eGFR, platelet count, potassium, sodium, and WBC; model 4, adjusted for model 3 and metformin, glimepiride, furosemide, beta-block, statin, CCB, ARB, and ACEI.

The ePVS_admission and ePVS_discharge were quartiled to better explore the trend for ePVS (**Supplementary Table S10**). The exact cutoff values for admission ePVS quartiles were Q1 [1.48, 4.31], Q2 (4.31, 4.98], Q3 (4.98, 5.90], and Q4 (5.90, 31.79]. The model 4 results (**Table 2**) indicated heterogeneous quartile-level effects. Notably, for the primary endpoint of CKM4, the Q2 stratum of admission ePVS provided no statistical evidence for elevated risk (HR = 1.04, *P >* 0.05); the significant excess risk was restricted to the Q3 and Q4 subgroups. Setting ePVS_admission Q1 as a reference (**Table 2**), the probabilities of progressing to CKM4, CKM4a, CKM4b, CAD, HF, and stroke in Q3 increased by 19%, 13%, 38%, 18%, 35%, and 16%, respectively. Setting ePVS_discharge Q1 as a reference, the probabilities of progressing to CKM4, CKM4a, CKM4b, CAD, HF, and stroke in Q4 increased by 43%, 22%, 88%, 40%, 76%, and 38%, respectively.

**Table 2:** Cox analysis for ePVS quartiles at various endpoints of model 4.

Endpoints	Characteristic	ePVS_admission		ePVS_discharge	
		HR (95% CI)	*P*-value	HR (95% CI)	*P*-value
CKM4	Q1	1.00 (reference)	Ref	1.00 (reference)	ref
	Q2	1.04 (0.99, 1.09)	0.11	1.06 (1.02, 1.11)	0.008
	Q3	1.19 (1.13, 1.25)	<0.001	1.17 (1.12, 1.23)	<0.001
	Q4	1.43 (1.36, 1.50)	<0.001	1.35 (1.28, 1.42)	<0.001
CKM4a	Q1	1.00 (reference)	Ref	1.00 (reference)	ref
	Q2	1.00 (0.95, 1.05)	>0.9	1.04 (0.99, 1.09)	0.11
	Q3	1.13 (1.07, 1.19)	<0.001	1.10 (1.04, 1.16)	<0.001
	Q4	1.22 (1.15, 1.29)	<0.001	1.17 (1.10, 1.24)	<0.001
CKM4b	Q1	1.00 (reference)	Ref	1.00 (reference)	ref
	Q2	1.13 (1.05, 1.22)	<0.001	1.19 (1.10, 1.28)	<0.001
	Q3	1.38 (1.28, 1.48)	<0.001	1.42 (1.32, 1.53)	<0.001
	Q4	1.88 (1.74, 2.03)	<0.001	1.73 (1.60, 1.88)	<0.001
CAD	Q1	1.00 (reference)	Ref	1.00 (reference)	ref
	Q2	1.01 (0.95, 1.07)	0.8	1.04 (0.98, 1.11)	0.2
	Q3	1.18 (1.11, 1.26)	<0.001	1.10 (1.03, 1.17)	0.003
	Q4	1.40 (1.30, 1.50)	<0.001	1.22 (1.13, 1.31)	<0.001
HF	Q1	1.00 (reference)	Ref	1.00 (reference)	ref
	Q2	1.11 (1.02, 1.20)	0.012	1.11 (1.03, 1.20)	0.009
	Q3	1.35 (1.24, 1.46)	<0.001	1.26 (1.16, 1.36)	<0.001
	Q4	1.76 (1.61, 1.92)	<0.001	1.50 (1.37, 1.64)	<0.001
Stroke	Q1	1.00 (reference)	Ref	1.00 (reference)	ref
	Q2	0.99 (0.90, 1.08)	0.8	1.03 (0.94, 1.12)	0.5
	Q3	1.16 (1.06, 1.27)	0.001	1.13 (1.03, 1.24)	0.008
	Q4	1.38 (1.25, 1.52)	<0.001	1.21 (1.09, 1.34)	<0.001

Quartile 1 (Q1) serves as the reference category. Quartile cutoff values for admission ePVS: Q1 [1.48, 4.31], Q2 (4.31, 4.98], Q3 (4.98, 5.90], and Q4 (5.90, 31.79] mL/g. Quartile cutoff values for discharge ePVS: Q1 [2.22, 4.68], Q2 (4.68, 5.54], Q3 (5.54, 6.72], and Q4 (6.72, 14.89] mL/g.

HR, hazard ratio; CI, confidence interval; ePVS, estimated plasma volume status; CKM4, cardiovascular-kidney-metabolic stage 4; CAD, coronary artery disease; HF, heart failure.

The exact cutoff values for discharge ePVS quartiles were Q1 [2.22, 4.68], Q2 (4.68, 5.54], Q3 (5.54, 6.72], and Q4 (6.72, 14.89]. Similar trends were observed for ePVS_discharge (**Table 2**), although a significant risk signal emerged starting from Q2 (HR = 1.06, *P* < 0.01) for the CKM4 endpoint. This indicates cross-time-point and cross-endpoint quartile-level heterogeneities between admission and discharge measurements. When ePVS_discharge Q1 was used as a reference, the probabilities of progressing to CKM4, CKM4a, CKM4b, CAD, HF, and stroke in Q3 increased by 17%, 10%, 42%, 10%, 26%, and 13%, respectively. Setting ePVS_admission Q1 as a reference, the probabilities of progressing to CKM4, CKM4a, CKM4b, CAD, HF, and stroke in Q4 increased by 35%, 17%, 73%, 22%, 50%, and 21%, respectively.

In the quartile-level analysis, we observed distinct heterogeneity in risk stratification across different time points and clinical endpoints. For the primary endpoint CKM4, admission ePVS demonstrated a threshold effect: compared to the Q1 reference, the Q2 stratum showed no statistical evidence of elevated risk (HR = 1.04, 95% CI: 0.99–1.09, *P* = 0.11), with significant excess risk restricted to the higher Q3 and Q4 strata (*P* < 0.001). Conversely, for discharge ePVS evaluated for the identical CKM4 endpoint, a significant risk signal emerged earlier, reaching statistical significance in the Q2 stratum (HR = 1.06, 95% CI: 1.02–1.11, *P* = 0.008). Similar cross-endpoint heterogeneities were observed. While volume-dependent endpoints such as heart failure and CKM4b exhibited a clear step-wise separation primarily in higher admission quartiles, endpoints driven by vascular damage, such as stroke, displayed divergent quartile effect patterns.

For the primary CKM4 endpoint, the Q4 association was numerically stronger for admission than for discharge ePVS (HR 1.43 vs. 1.35).When Q1 was set as a reference (**Table 2**), the CKM4 risk of Q4 increased by 43% and 35% in ePVS_admission and ePVS_discharge, respectively. The CKM4b risk of Q4 increased by 88% and 73% in ePVS_admission and ePVS_discharge, respectively. Even though both ePVS_admission and ePVS_discharge were correlated with the risk of CKM4, ePVS_admission was more suitable for hierarchical management. However, this finding still requires external validation in independent cohorts before admission ePVS can be recommended for hierarchical risk management.

### 3.4 Effects of the interaction effect of age and sex on the Cox analysis for ePVSs and endpoints

Significant age interactions were observed for CKM4, CKM4a, CKM4b, and CAD for both admission and discharge ePVS and for HF only for admission ePVS; no significant age interaction was observed for stroke (**Table 3**). Moreover, ePVS_discharge and ePVS_admission indicated no interaction for sex. In the ePVS_admission quartiles (**Supplementary Table S11**), patients aged >60 years had a higher HR than those aged ≤60 years in terms of CKM4 and CKM4a risk. Men had a higher HR than women did for CKM4b, CAD, and HF risk. In the ePVS_admission quartiles (**Supplementary Table S11**), patients aged >60 years had a higher HR than those aged ≤60 years in terms of CKM4 and CKM4a risk. Men had a higher HR than women did for CKM4b, CAD, and HF risk. In the ePVS_discharge quartiles (**Supplementary Table S11**), patients aged >60 years had a higher hazard ratio (HR) than those aged ≤60 years in the CKM4 and CKM4a quartiles and a higher CAD risk. Men had a greater HR than women did in CKM4b.

**Table 3:** The interaction effect of age and sex influences the ePVS result of model 4.

Endpoint	Subgroup	ePVS_admission			ePVS_discharge		
		HR (95% CI)	*P*-value	*P* for interaction	HR (95% CI)	*P*-value	*P* for interaction
CKM4	Sex			0.28			0.699
	F	1.08 (1.06, 1.09)	<0.001		1.08 (1.06, 1.10)	<0.001	
	M	1.07 (1.06, 1.09)	<0.001		1.09 (1.07, 1.11)	<0.001	
	Age			< 0.001			0.008
	>60	1.08 (1.07, 1.10)	<0.001		1.08 (1.07, 1.10)	<0.001	
	≤60	1.07 (1.05, 1.08)	<0.001		1.08 (1.06, 1.10)	<0.001	
CKM4a	Sex			0.408			0.55
	F	1.05 (1.04, 1.07)	<0.001		1.04 (1.02, 1.06)	<0.001	
	M	1.05 (1.03, 1.06)	<0.001		1.05 (1.03, 1.07)	<0.001	
	Age			0.001			0.025
	>60	1.05 (1.04, 1.07)	<0.001		1.04 (1.02, 1.06)	<0.001	
	≤60	1.04 (1.02, 1.06)	<0.001		1.04 (1.02, 1.06)	<0.001	
CKM4b	Sex			0.114			0.114
	F	1.09 (1.07, 1.11)	<0.001		1.09 (1.07, 1.11)	<0.001	
	M	1.10 (1.08, 1.11)	<0.001		1.10 (1.08, 1.11)	<0.001	
	Age			< 0.001			< 0.001
	>60	1.11 (1.09, 1.13)	<0.001		1.11 (1.09, 1.13)	<0.001	
	≤60	1.09 (1.07, 1.10)	<0.001		1.09 (1.07, 1.10)	<0.001	
CAD	Sex			0.921			0.717
	F	1.07 (1.05, 1.09)	<0.001		1.05 (1.02, 1.07)	<0.001	
	M	1.08 (1.06, 1.09)	<0.001		1.08 (1.05, 1.10)	<0.001	
	Age			< 0.001			0.003
	>60	1.09 (1.07, 1.11)	<0.001		1.07 (1.05, 1.09)	<0.001	
	≤60	1.06 (1.04, 1.08)	<0.001		1.04 (1.02, 1.07)	0.002	
HF	Sex			0.301			0.778
	F	1.08 (1.06, 1.11)	<0.001		1.10 (1.07, 1.13)	<0.001	
	M	1.11 (1.09, 1.13)	<0.001		1.14 (1.11, 1.17)	<0.001	
	Age			0.009			0.288
	>60	1.12 (1.09, 1.14)	<0.001		1.11 (1.09, 1.14)	<0.001	
	≤60	1.09 (1.07, 1.12)	<0.001		1.11 (1.07, 1.15)	<0.001	
Stroke	Sex			0.604			0.678
	F	1.08 (1.05, 1.10)	<0.001		1.05 (1.02, 1.09)	0.003	
	M	1.07 (1.04, 1.10)	<0.001		1.05 (1.02, 1.09)	0.003	
	Age			0.37			0.857
	>60	1.07 (1.04, 1.09)	<0.001		1.03 (1.00, 1.07)	0.026	
	≤60	1.07 (1.04, 1.10)	<0.001		1.08 (1.03, 1.12)	<0.001	

### 3.5 RCS analysis to explore the overall trend of ePVSs

The RCS plots of ePVS_admission were applied at various endpoints (**Figure 5**). All of the endpoints were nonlinear with a spoon-shaped curve, exhibiting a distinct threshold pattern: the risk curve remained relatively flat at lower ePVS concentrations and increased steeply once ePVS exceeded a critical value. In model 4, the clinical inflection points were numerically similar but varied slightly across endpoints, ranging from 5.136 to 5.151 (**Supplementary Table S12**).

**Figure 5:**
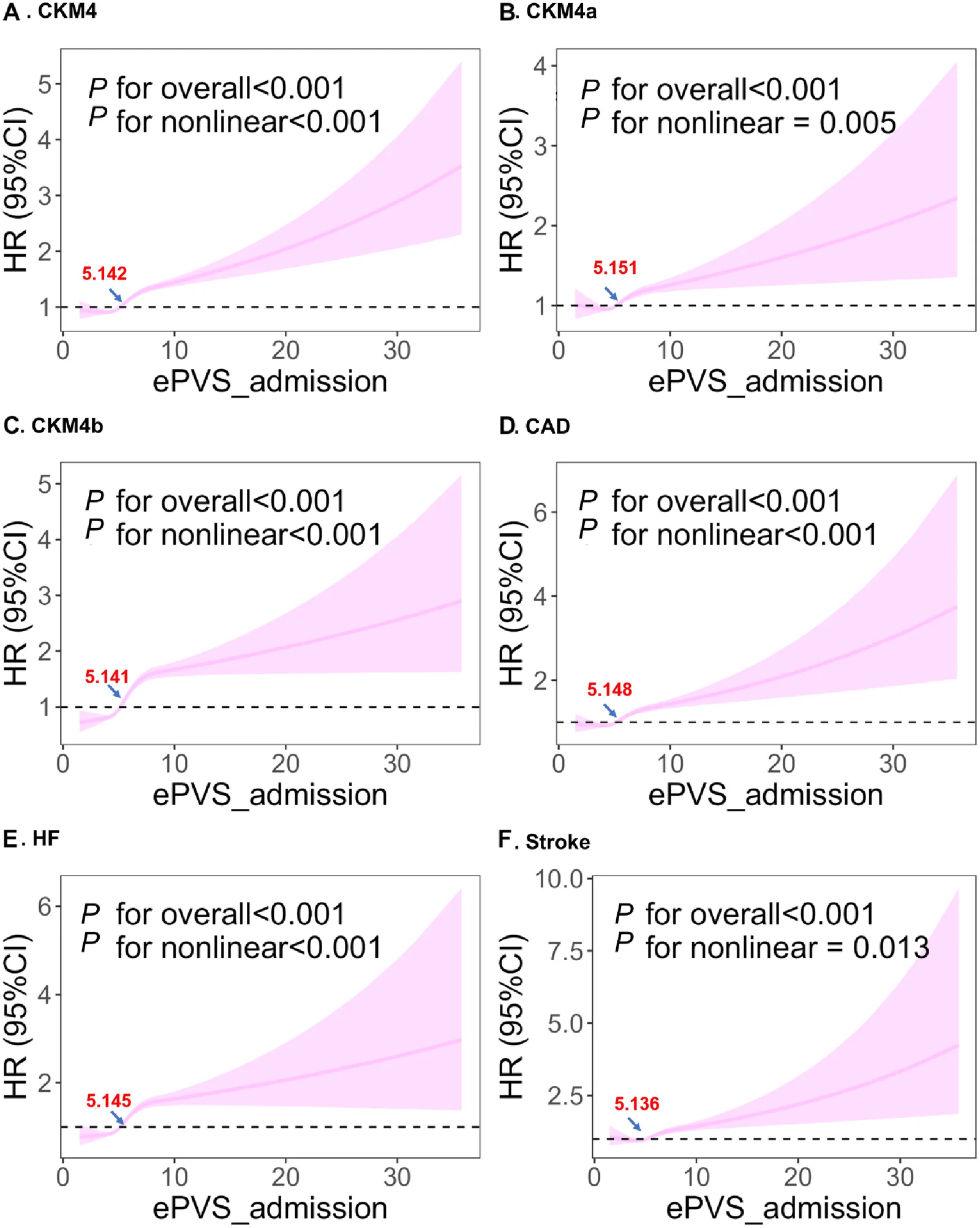
Restricted cubic spline analyses demonstrating the nonlinear association between admission ePVS and the hazard of each endpoint. **(A)** CKM4 progression; **(B)** CKM4a progression; **(C)** CKM4b progression; **(D)** coronary artery disease (CAD); **(E)** heart failure (HF); **(F)** stroke. The solid pink line represents the adjusted hazard ratio (HR), and the light pink shaded area indicates the 95% confidence interval (CI). P values for overall association and nonlinear effect are presented in each panel. The dashed horizontal line marks HR = 1, and the blue arrow indicates the reference value of ePVS.

For ePVS_discharge (**Supplementary Figure S2**), significant overall associations were observed across all endpoints. Significant nonlinear associations were identified for CKM4 and CAD, whereas the remaining endpoints showed approximately linear associations. In model 4, the clinical inflection point ranged from 5.775 to 5.853 across endpoints (**Supplementary Table S12**).

### 3.6 Cumulative risk curve of ePVSs

The RCS indicated that the clinical inflection points of ePVS_admission and ePVS_discharge were approximately 5.14 and 5.78, respectively. Given that the independently estimated inflection points for all secondary endpoints were numerically very close to these values (**Supplementary Table S14**), we applied these primary CKM4-derived cutoffs as uniform exploratory thresholds across all analyses. In the ePVS_admission quartiles, Q4 had a much greater risk than did Q1–Q3 (**Figure 6**), especially in CKM4b and HF. Similar trends were shown in ePVS_discharge quartiles (**Supplementary Figure S5**), and CKM4b clearly tended to separate in Q1–Q4. Patients with ePVS_admission >5.14 had a greater risk for all endpoints (**Supplementary Figure S4**) than those with ePVS_admission ≤5.14. Patients with ePVS_discharge >5.78 had a greater risk for all endpoints (**Supplementary Figure S5**) than those with ePVS_discharge ≤5.78.

**Figure 6:**
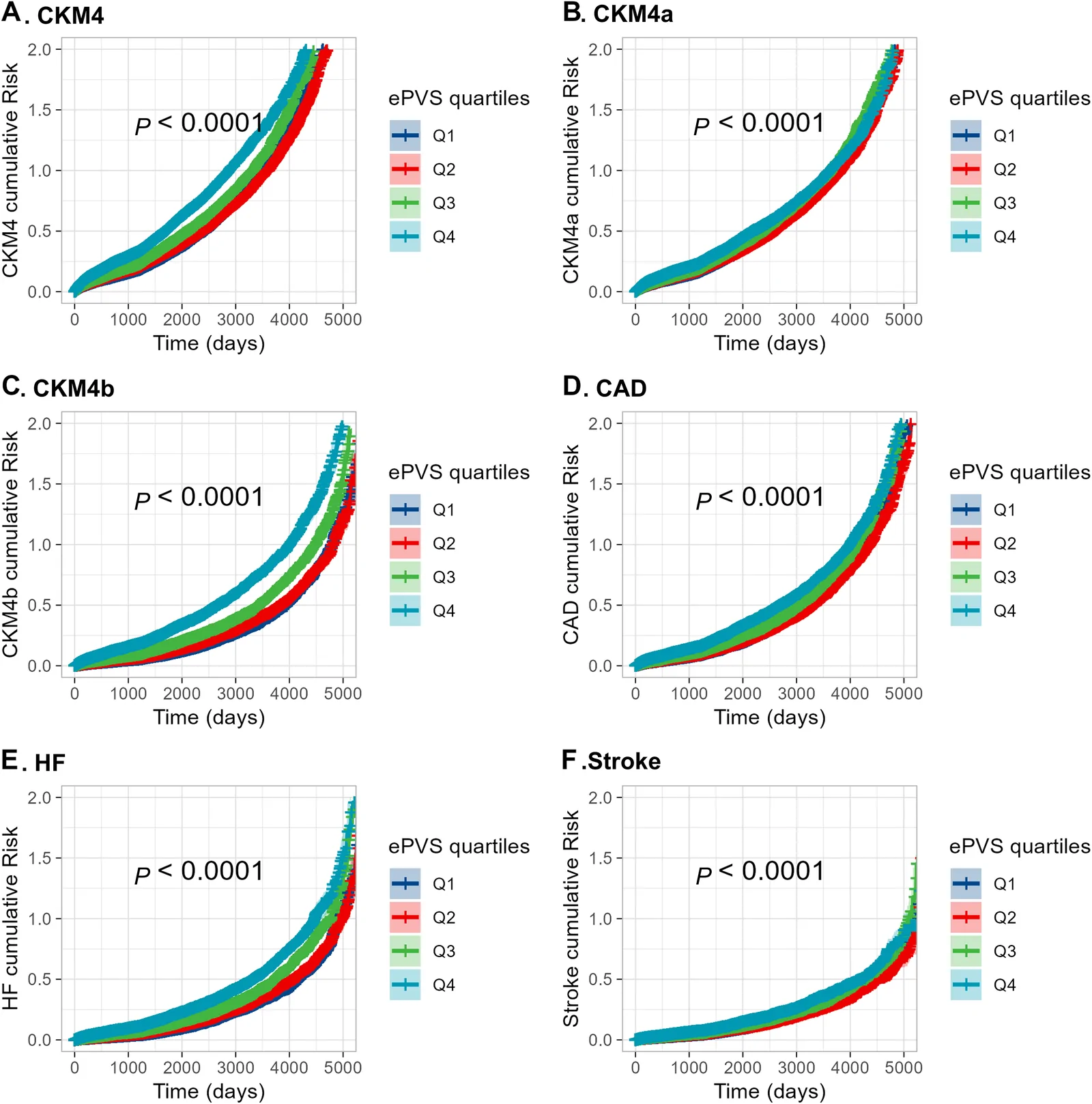
Cumulative risk curves stratified by admission ePVS quartiles across all study endpoints. **(A)** CKM4 progression; **(B)** CKM4a progression; **(C)** CKM4b progression; **(D)** coronary artery disease (CAD); **(E)** heart failure (HF); **(F)** stroke. Q1 to Q4 correspond to the first to fourth quartiles of admission ePVS, respectively. P values from the log-rank test are shown in each panel.

### 3.7 Sensitivity analysis

For the primary CKM4 endpoint, the direction and significance pattern of the quartile associations were generally consistent in the parsimonious model. Some variation in effect magnitude and statistical significance was observed for secondary endpoints, indicating that these secondary findings should be interpreted cautiously (**Supplementary Table S13**).

We further assessed the robustness of our findings using Fine–Gray competing risk models. Because patients with severe volume overload may die before developing kidney failure, all-cause mortality during follow-up was treated as the primary competing event. The results were consistent with the main analysis. These findings indicate that the standard Cox regression results were not materially biased by competing risks (**Supplementary Table S14**).

## 4 Discussion

To the best of our knowledge, this is the first study to investigate the association between estimated plasma volume status and the risk of progression from CKM stage 2 to stage 4. This study answered four questions. First, ePVS (both at admission and at discharge) is associated with progression from CKM2 to CKM4, but they exhibited distinct risk stratification profiles. Admission ePVS showed a threshold-type nonlinear association with CKM4 progression, characterized by a spoon-shaped curve, with significant risk elevation restricted to the upper quartiles. Among patients with higher ePVS, the association with several outcomes was stronger in those aged >60 years.

Why are ePVSs at admission more suitable for CKM4 risk stratification? Our results revealed that even ePVSs at discharge were correlated with endpoints such as CKM4, CKM4a, CKM4b, CAD, HF, and stroke, but the quartile risk stratification was less than that of ePVS admission quartiles. Three reasons might have led to this result. First, the ePVS at admission with no pharmacological intervention might be more suitable for characterizing the baseline pathological condition. Second, even though the ePVS of discharge has been human-induced, it has approached a normal level, which fails to represent the long-term capacity status. Numerous interventions, such as (15) dapagliflozin and diuretics, have been confirmed to be correlated with ePVSs (16). After discharge, it is prone to revert to the original condition. Therefore, the ePVSs at discharge were changed to drugs or other interventions that are not suitable for characterizing the initial status. Third, stroke (especially cardioembolic stroke and atherosclerotic stroke) is usually driven by long-term vascular damage (such as carotid plaque) (17) rather than short-term volume fluctuations. In contrast, HF (18) and kidney failure (19) are more directly related to the volume status. Therefore, the stratified accumulation risk of ePVSs at admission shows a more significant separation trend between HFs and CKM4b.

Another question is, when ePVS increased in CKM2 patients, why did patients >60 years old have a greater risk of endpoints (CKM4, CKM4a, CKM4b, CAD, and HF) than those ≤60 years old? The plasma volume may decrease in older patients because of a reduction in muscle mass (20), impaired renal concentrating ability (21), and deterioration of cardiovascular function (e.g., reduced cardiac output may affect the circulating blood volume). The abovementioned factors are closely linked to a decrease in the plasma volume of older patients. Higher plasma volume may reflect greater cardiovascular and renal hemodynamic burden on the cardiovascular system and kidney, which correlates with the progression of CKM4. The age-related risk differences in ePVS CKM4 patients provide a basis for the stratified management of CKM4 patients.

The ePVS (both at admission and at discharge) may have potential prognostic value in patients with CKM2. We processed the quartiles of ePVS_admission in model 4 (**Table 2**) and set Q1 as a reference; Q3 and Q4 were associated with a 19% and 43% higher probability of progressing to CKM4, respectively. In the quartile of ePVS_discharge in model 4 (**Table 2**), Q1 was set as a reference; Q2, Q3, and Q4 increased by 6%, 17%, and 35%, respectively, in probability to CKM4. In addition, especially for CKM4b and HF, Q2, Q3, and Q4 associated with higher hazards for CKM4b by 13%, 38%, and 88%, respectively, and for HF by 11%, 35%, and 76%, respectively. For the other endpoints (CKM4a, CAD, and stroke), similar trends were observed.

Underlying diseases associated with CKM2 may contribute to higher ePVS. We defined CKM2 patients as patients with DM, hypertension, MetS, hypertriglyceridemia, or nonhypertriglyceridemia but with serum triglyceride levels ≥135 mg/dL. Hypertension patients might have increased sodium intake (22, 23), and sodium is the main determinant of plasma osmotic pressure. When sodium is retained, water is retained simultaneously to maintain isotonicity. In other words, the more severe the hypertension is, the greater the plasma volume. Similarly, increasing circulating glucose and triglyceride levels increase the plasma osmotic pressure and promote the retention of water. In DM, insulin resistance (24) promotes sodium and water retention, which increases the plasma volume. In hyperlipidemia, circulating lipids can also damage the vascular endothelium (25, 26), increase the permeability of the endothelium, and increase the plasma volume. In summary, the plasma volume is closely related to the pathogenesis of CKM2; higher ePVS may indicate more severe CKM2.

Notably, the RCS curve for admission ePVS showed a nonlinear pattern in which the overall significant association was driven predominantly by risk elevation in the upper exposure range. This pattern aligns with the hypothesis that baseline intrinsic plasma volume overload must exceed a critical level to substantially contribute to CKM progression, and moderate elevations within the Q2 range do not translate to measurable prognostic harm.

In contrast, discharge ePVS paired with more linear dose–response profiles for most endpoints. This divergence is likely attributable to the modification of discharge ePVS by in-hospital volume management, including diuretic administration and SGLT-2 inhibitor initiation. Accordingly, discharge ePVS may reflect treatment-modified volume status rather than pure baseline pathophysiology, and the Q2 signal for discharge ePVS may be partially influenced by clinical interventions rather than solely reflecting intrinsic baseline volume status. Collectively, these findings do not support a uniform linear risk association across the full spectrum of ePVS, and modest elevations in baseline ePVS cannot be assumed to independently increase the risk of CKM deterioration.

These effect sizes are highly comparable to recent findings in overt cardiovascular disease cohorts—for instance, in patients with myocardial infarction complicated by HF, the highest quartile of ePVS had a 51% increased risk (HR = 1.51) for 1-year mortality (27). Similarly, a high ePVS (>5.5 mL/g) at discharge was independently associated with 88% higher risk of all-cause mortality (HR = 1.879) in cardiovascular patients. Regarding kidney-related outcomes (28), in severe acute kidney injury, a per-unit increase in ePVS correlated with a robustly elevated risk of adverse outcomes (HR = 1.05) and impaired renal recovery (29). The fact that the magnitude of risk in our relatively early-stage CKM2 population is comparable to those observed in established single-organ disease cohorts strongly reinforces the clinical value of ePVS.

This work has three strengths: first, we explored the relationship between the plasma volume and the progression of CKM2 to CKM4; second, sufficient confounding factors were included to adjust the results; and third, the sample size and follow-up period were sufficient. Previous studies focused only on the association between insulin resistance and CKM (30, 31) but ignored the plasma volume. Previous studies have set mortality as the endpoint (32) but not CKM4, which is not conducive to analyzing the process of CKM progression.

From a clinical perspective, our findings suggest that ePVS may provide additional information for risk stratification among patients with CKM stage 2. Patients with higher admission ePVS values, particularly those within the upper quartile range (Q3: 4.98–5.90 mL/g), may represent a subgroup with greater cardiovascular and metabolic burden and may benefit from closer evaluation of volume status and comprehensive risk management. Furthermore, the inflection points identified by restricted cubic spline analyses suggest potential threshold effects in the association between ePVS and CKM progression, providing additional insights into the identification of individuals at higher risk. For patients with ePVS values exceeding the upper quartile cutoff (Q4: >5.90 mL/g) or approaching the nonlinear inflection point (5.14 mL/g), ePVS may serve as a useful marker to facilitate clinical risk assessment and individualized management. Similarly, persistently elevated discharge ePVS (>6.72 mL/g) may indicate a higher residual volume-related burden and help identify patients who may require closer follow-up after discharge. Further studies are needed to determine whether incorporating ePVS-based risk stratification into clinical practice can improve patient management and outcomes.

This study has several important limitations (30). First, as a surrogate biomarker calculated from hemoglobin and hematocrit rather than a direct measurement of plasma volume, ePVS carries inherent measurement noise and may not perfectly reflect actual intravascular volume status, which could attenuate the precision of observed effect estimates. Second, given the observational study design, reverse causality cannot be definitively excluded. Elevated plasma volume status may be a consequence of progressive CKM pathophysiology rather than a causal driver of disease progression (33). Accordingly, our findings demonstrate prognostic associations but do not imply that modifying ePVS levels would directly improve CKM-related clinical outcomes. Third, despite extensive adjustment for established demographic, clinical, and treatment-related covariates, residual unmeasured confounding cannot be fully eliminated, which may partially contribute to the heterogeneous effect estimates observed across time points and endpoints (34, 35). Fourth, this cohort was recruited from a single regional center in Tianjin, China, and no external validation was performed in independent populations. Therefore, the generalizability of ePVS as a prognostic biomarker for CKM progression remains unclear, and our findings require replication in multi-center cohorts and diverse ethnic groups. Finally, ePVS was only measured at two time points (admission and discharge), which may introduce misclassification bias and bias the results toward the null due to regression dilution effects.

## 5 Conclusion

Higher admission ePVS (Q3/Q4) was independently associated with the progression from CKM2 to CKM4, whereas moderate elevation (Q2) showed no significant risk, which does not support the inference that mild-to-moderate ePVS elevation automatically increases CKM-progression risk. Admission ePVS, being less confounded by in-hospital drug influences, appears more suitable for risk stratification. However, these findings should be considered hypothesis-generating. External-cohort validation of biomarker performance is required before establishing its clinical utility for risk assessment or volume management in CKM patients.

## Data Availability

The datasets presented in this article are not readily available because The TJFHIT is not in a public database, but the primary data can be obtained by contacting the authors. Requests to access the datasets should be directed to xuqiangtjutcm1973@outlook.com.
