# Supplementary material for: Association of estimated plasma volume status with progression from stage 2 to stage 4 cardiovascular–kidney–metabolic syndrome: a cohort from the Tianjin region of China
Source: Front Endocrinol (Lausanne). 2026 Oct 15;17:1959815. doi: 10.3389/fendo.2026.1959815 (PMC13716659; doi:10.3389/fendo.2026.1959815)
Supplement: Supplementary file 1 [file SupplementaryFile1.docx]

**Association of estimated plasma volume status with progression from stage 2 to stage 4 cardiovascular–kidney–metabolic syndrome: a cohort from the Tianjin region of China**

**Content**

[Table S1. Variance inflation factor (VIF) results.	4](#_Toc23354)

[Table S2. Missing data proportions for study variables	6](#_Toc10683)

[Table S3. Baseline characteristics of CKM2 patients who progress to CKM4a or non-CKM4a	7](#_Toc23954)

[Table S4. Baseline characteristics of CKM2 patients who progress to CKM4b or non-CKM4b	10](#_Toc22127)

[Table S5. Baseline characteristics of CKM2 patients who progress to CAD or non-CAD	13](#_Toc5983)

[Table S6. Baseline characteristics of CKM2 patients who progress to HF or non-HF	16](#_Toc22500)

[Table S7. Baseline characteristics of CKM2 patients who progress to stroke or non-stroke	19](#_Toc14981)

[Table S8. Mean absolute SHAP values for the six endpoints.	22](#_Toc23431)

[Table S9. OOB prediction error and discriminative performance of the random forest models used for SHAP analysis across various endpoints.	24](#_Toc15748)

[Table S10. The Cox analysis for ePVS quartiles in various endpoints	25](#_Toc10610)

[Table S11. The interaction of age and sex influences the ePVS quartiles result of Model 4	32](#_Toc19752)

[Table S12. Estimated cut-off points and 95% confidence intervals for admission and discharge ePVS across different endpoints.	38](#_Toc5112)

[Table S13. Associations between ePVS quartiles and clinical outcomes in the parsimonious model	39](#_Toc12387)

[Table S14. Fine-Gray competing risk models for progression to CKM stage 4b	40](#_Toc13442)

[Figure S1. The forest plot for ePVS_discharge in five models. (Model 0: unadjusted; Model 1: adjusted with sex, age, and LOS; Model 2: adjusted with Model 1 and DM, hypertension, hypertriglyceridemia, MetS, CKD, anemia, hyperuricemia; Model 3: adjusted with Model 2 and bicarbonate, chloride, eGFR, platelet count, potassium, sodium, and WBC; Model 4: adjusted with Model 3 and metformin, glimepiride, furosemide, beta-block, statin, CCB, ARB, and ACEI)	42](#_Toc22577)

[Figure S2. The RCS analysis for various endpoints with the ePVS_discharge	43](#_Toc26735)

[Figure S3. The Cumulative Risk Curve for various endpoints with ePVS_discharge quartiles	44](#_Toc25474)

[Figure S4. The Cumulative Risk Curve for various endpoints with ePVS_admission > 5.14 or not	45](#_Toc21972)

[Figure S5. The Cumulative Risk Curve for various endpoints with ePVS_discharge >5.78 or not	46](#_Toc24434)

# Table S1. Variance inflation factor (VIF) results.

| **Exposure** | **Characteristic** | **VIF** |
| --- | --- | --- |
| **ePVS_admission** | Sex | 1.33 |
|  | Age | 1.29 |
|  | LOS | 1.00 |
|  | DM | 1.47 |
|  | Hypertension | 1.75 |
|  | Hypertriglyceridemia | 1.10 |
|  | MetS | 1.01 |
|  | CKD | 1.21 |
|  | Anemia | 1.27 |
|  | Hyperuricemia | 1.04 |
|  | Bicarbonate | 1.04 |
|  | eGFR | 1.28 |
|  | PlateletCount | 1.11 |
|  | Potassium | 1.10 |
|  | WBC | 1.00 |
|  | Chloride | 1.14 |
|  | Sodium | 1.27 |
|  | Metformin | 1.34 |
|  | Glimepiride | 1.14 |
|  | Furosemide | 1.17 |
|  | beta-Blocker | 1.07 |
|  | Statin | 1.15 |
|  | CCB | 1.39 |
|  | ARB | 1.27 |
|  | ACEI | 1.22 |
| **ePVS_discharge** | Sex | 1.36 |
|  | Age | 1.32 |
|  | LOS | 1.00 |
|  | DM | 1.47 |
|  | Hypertension | 1.75 |
|  | Hypertriglyceridemia | 1.11 |
|  | MetS | 1.01 |
|  | CKD | 1.22 |
|  | Anemia | 1.26 |
|  | Hyperuricemia | 1.04 |
|  | Bicarbonate | 1.04 |
|  | eGFR | 1.28 |
|  | Platelet Count | 1.11 |
|  | Potassium | 1.10 |
|  | WBC | 1.00 |
|  | Chloride | 1.13 |
|  | Sodium | 1.25 |
|  | Metformin | 1.34 |
|  | Glimepiride | 1.14 |
|  | Furosemide | 1.16 |
|  | beta-Blocker | 1.07 |
|  | Statin | 1.15 |
|  | CCB | 1.39 |
|  | ARB | 1.27 |
|  | ACEI | 1.22 |

#LOS, length of stay; DM, diabetes mellitus; MetS, metabolic syndrome; CKD, chronic kidney diseases; ePVS_admission, estimated plasma volume statute at admission; ePVS_discharge, estimated plasma volume statute at discharge; eGFR, estimated glomerular filtration rate; WBC, white blood cell count; CCB, calcium channel blockers; ARB, angiotensin II receptor blocker; ACEI, angiotensin-converting enzyme inhibitor.

# Table S2. Missing data proportions for study variables

| **Characteristic** | **N** | **Missing n(%)** |
| --- | --- | --- |
| **ePVS_admission** | 36505 | 0 |
| **ePVS_discharge** | 36505 | 0 |
| **Sex** | 36505 | 2(0.01%) |
| **Age** | 36505 | 0 |
| **LOS** | 36505 | 0 |
| **DM** | 36505 | 0 |
| **Hypertension** | 36505 | 0 |
| **Hypertriglyceridemia** | 36505 | 0 |
| **MetS** | 36505 | 0 |
| **CKD** | 36505 | 0 |
| **Anemia** | 36505 | 0 |
| **Hyperuricemia** | 36505 | 0 |
| **Bicarbonate** | 36505 | 1657 (4.54%) |
| **eGFR** | 36505 | 1378(3.77%) |
| **Platelet Count** | 36505 | 1376 (3.77%) |
| **Potassium** | 36505 | 1150 (3.15%) |
| **WBC** | 36505 | 1116 (3.06%) |
| **Chloride** | 36505 | 1436 (3.93%) |
| **Sodium** | 36505 | 1743(4.77%) |
| **Metformin** | 36505 | 1683(4.61%) |
| **Glimepiride** | 36505 | 1739(4.76%) |
| **Furosemide** | 36505 | 1737 (4.76%) |
| **beta-Blocker** | 36505 | 1342 (3.68%) |
| **Statin** | 36505 | 87 (0.24%) |
| **CCB** | 36505 | 1599 (4.38%) |
| **ARB** | 36505 | 421 (1.15%) |
| **ACEI** | 36505 | 1176 (3.22%) |

#LOS, length of stay; DM, diabetes mellitus; MetS, metabolic syndrome; CKD, chronic kidney diseases; ePVS_admission, estimated plasma volume statute at admission; ePVS_discharge, estimated plasma volume statute at discharge; eGFR, estimated glomerular filtration rate; WBC, white blood cell count; ACEI, angiotensin-converting enzyme inhibitor.

# Table S3. Baseline characteristics of CKM2 patients who progress to CKM4a or non-CKM4a

| **Characteristic** | **Overall N = 36,505** | **Non-CKM4a N = 24,222** | **CKM4a N = 12,283** | ***P* value** |
| --- | --- | --- | --- | --- |
| **Demographic** |  |  |  |  |
| **Sex (Male), n (%)** | 16,388 (45%) | 10,684 (44%) | 5,704 (46%) | <0.001 |
| **Age, years** | 61 (50, 72) | 59 (46, 70) | 66 (56, 76) | <0.001 |
| **LOS, days** | 3.0 (1.0, 5.0) | 3.0 (1.0, 6.0) | 2.0 (1.0, 5.0) | <0.001 |
| **Medical history** |  |  |  |  |
| **DM, n (%)** | 11,780 (32%) | 7,601 (31%) | 4,179 (34%) | <0.001 |
| **Hypertension, n (%)** | 25,391 (70%) | 16,291 (67%) | 9,100 (74%) | <0.001 |
| **Hypertriglyceridemia, n (%)** | 1,670 (4.6%) | 1,174 (4.8%) | 496 (4.0%) | <0.001 |
| **MetS, n (%)** | 65 (0.2%) | 55 (0.2%) | 10 (<0.1%) | 0.002 |
| **CKD, n (%)** | 11,926 (33%) | 7,852 (32%) | 4,074 (33%) | 0.15 |
| **Anemia, n (%)** | 9,169 (25%) | 6,423 (27%) | 2,746 (22%) | <0.001 |
| **Hypothyroidism, n(%)** | 4,128 (11%) | 2,696 (11%) | 1,432 (12%) | 0.13 |
| **Hyperuricemia, n(%)** | 1,475 (4.0%) | 978 (4.0%) | 497 (4.0%) | >0.9 |
| **Lab result** |  |  |  |  |
| **ePVS_admission, (mL/g), Median (IQR)** | 5.14 (4.39, 6.18) | 5.08 (4.36, 6.08) | 5.16 (4.40, 6.23) | <0.001 |
| **ePVS_discharge, (mL/g), Median (IQR)** | 5.77 (4.82, 7.11) | 5.68 (4.76, 6.93) | 5.83 (4.85, 7.21) | <0.001 |
| **eGFR, mL/(min·1.73m^2), Median (IQR)** | 75 (54, 97) | 76 (53, 100) | 73 (55, 92) | <0.001 |
| **Anion Gap, mEq/L, Median (IQR)** | 15.0 (13.0, 17.0) | 15.0 (13.0, 17.0) | 14.0 (13.0, 16.0) | <0.001 |
| **Bicarbonate, mEq/L, Median (IQR)** | 25.0 (23.0, 27.0) | 25.0 (22.0, 27.0) | 26.0 (23.0, 28.0) | <0.001 |
| **Chloride, mEq/L, Median (IQR)** | 102.0 (99.0, 105.0) | 102.0 (99.0, 105.0) | 102.0 (99.0, 105.0) | 0.8 |
| **Creatinine, mg/dL, Median (IQR)** | 0.90 (0.70, 1.20) | 0.90 (0.70, 1.30) | 1.00 (0.80, 1.20) | 0.075 |
| **FBG, mg/dL, Median (IQR)** | 116 (98, 148) | 115 (98, 147) | 117 (99, 150) | <0.001 |
| **Hematocrit, %, Median (IQR)** | 36.9 (32.9, 40.4) | 36.7 (32.7, 40.4) | 37.1 (33.2, 40.5) | <0.001 |
| **Hemoglobin, g/dL, Median (IQR)** | 12.30 (10.80, 13.60) | 12.20 (10.80, 13.60) | 12.40 (11.00, 13.60) | <0.001 |
| **MCHC, g/dL, Median (IQR)** | 33.30 (32.40, 34.30) | 33.30 (32.30, 34.30) | 33.40 (32.40, 34.40) | <0.001 |
| **MCH, pg, Median (IQR)** | 30.00 (28.50, 31.50) | 30.00 (28.50, 31.50) | 30.00 (28.50, 31.50) | 0.9 |
| **MCV, fL, Median (IQR)** | 90 (86, 94) | 90 (86, 94) | 90 (86, 94) | 0.090 |
| **Platelet Count, K/uL, Median (IQR)** | 237 (183, 301) | 237 (183, 301) | 235 (183, 299) | 0.7 |
| **Potassium, mEq/L, Median (IQR)** | 4.10 (3.80, 4.50) | 4.10 (3.80, 4.50) | 4.10 (3.80, 4.50) | 0.6 |
| **RDW, %, Median (IQR)** | 13.80 (13.10, 14.90) | 13.80 (13.10, 14.90) | 13.90 (13.20, 14.90) | <0.001 |
| **RBC, m/uL, Median (IQR)** | 4.13 (3.65, 4.56) | 4.11 (3.62, 4.56) | 4.15 (3.69, 4.57) | <0.001 |
| **Sodium, mEq/L, Median (IQR)** | 138.0 (136.0, 141.0) | 138.0 (136.0, 141.0) | 139.0 (136.0, 141.0) | 0.010 |
| **WBC, K/uL, Median (IQR)** | 8.6 (6.5, 11.4) | 8.7 (6.5, 11.6) | 8.4 (6.4, 11.0) | <0.001 |
| **Medication history** |  |  |  |  |
| **Metformin, n (%)** | 2,297 (6.3%) | 1,391 (5.7%) | 906 (7.4%) | <0.001 |
| **Glimepiride, n (%)** | 71 (0.2%) | 38 (0.2%) | 33 (0.3%) | 0.022 |
| **Furosemide, n (%)** | 4,963 (14%) | 3,141 (13%) | 1,822 (15%) | <0.001 |
| **beta-Block, n(%)** | 6,862 (19%) | 3,785 (16%) | 3,077 (25%) | <0.001 |
| **Statin, n (%)** | 5,654 (15%) | 3,279 (14%) | 2,375 (19%) | <0.001 |
| **CCB, n (%)** | 6,724 (18%) | 4,300 (18%) | 2,424 (20%) | <0.001 |
| **ARB, n (%)** | 3,276 (9.0%) | 1,958 (8.1%) | 1,318 (11%) | <0.001 |
| **ACEI, n(%)** | 8,918 (24%) | 5,335 (22%) | 3,583 (29%) | <0.001 |

\# LOS, length of stay; DM, diabetes mellitus; MetS, metabolic syndrome; CKD, chronic kidney diseases; ePVS_admission, estimated plasma volume statute at admission; ePVS_discharge, estimated plasma volume statute at discharge; eGFR, estimated glomerular filtration rate; FBG, fast blood glucose; MCHC, mean corpuscular hemoglobin concentration; MCH, mean corpuscular hemoglobin; MCV, mean cell volume; RDW, red blood cell distribution width; RBC, red blood cell count; WBC, white blood cell count; CCB, calcium channel blockers; ARB, angiotensin II receptor blocker; ACEI, angiotensin-converting enzyme inhibitor.

# Table S4. Baseline characteristics of CKM2 patients who progress to CKM4b or non-CKM4b

| **Characteristic** | **Overall N = 36,505** | **Non-CKM4b N = 29,421** | **CKM4b N = 7,084** | ***P* value** |
| --- | --- | --- | --- | --- |
| **Demographic** |  |  |  |  |
| **Sex (Male), n (%)** | 16,388 (45%) | 12,735 (43%) | 3,653 (52%) | <0.001 |
| **Age, years** | 61 (50, 72) | 60 (48, 71) | 66 (56, 77) | <0.001 |
| **LOS, days** | 3.0 (1.0, 5.0) | 3.0 (1.0, 5.0) | 3.0 (1.0, 5.0) | <0.001 |
| **Medical history** |  |  |  |  |
| **DM, n (%)** | 11,780 (32%) | 8,717 (30%) | 3,063 (43%) | <0.001 |
| **Hypertension, n (%)** | 25,391 (70%) | 21,202 (72%) | 4,189 (59%) | <0.001 |
| **Hypertriglyceridemia, n (%)** | 1,670 (4.6%) | 1,399 (4.8%) | 271 (3.8%) | <0.001 |
| **MetS, n(%)** | 65 (0.2%) | 58 (0.2%) | 7 (<0.1%) | 0.078 |
| **CKD, n (%)** | 11,926 (33%) | 8,031 (27%) | 3,895 (55%) | <0.001 |
| **Anemia, n (%)** | 9,169 (25%) | 6,973 (24%) | 2,196 (31%) | <0.001 |
| **Hypothyroidism, n (%)** | 4,128 (11%) | 3,272 (11%) | 856 (12%) | 0.022 |
| **Hyperuricemia, n (%)** | 1,475 (4.0%) | 996 (3.4%) | 479 (6.8%) | <0.001 |
| **Lab result** |  |  |  |  |
| **ePVS_admission, (mL/g), Median (IQR)** | 5.14 (4.39, 6.18) | 5.06 (4.35, 6.06) | 5.48 (4.58, 6.63) | <0.001 |
| **ePVS_discharge, (mL/g), Median (IQR)** | 5.77 (4.82, 7.11) | 5.68 (4.76, 7.00) | 6.17 (5.08, 7.51) | <0.001 |
| **eGFR, mL/(min·1.73m^2), Median (IQR)** | 75 (54, 97) | 79 (58, 102) | 56 (37, 77) | <0.001 |
| **Anion Gap, mEq/L, Median (IQR)** | 15.0 (13.0, 17.0) | 14.0 (12.0, 17.0) | 15.0 (13.0, 17.0) | <0.001 |
| **Bicarbonate, mEq/L, Median (IQR)** | 25.0 (23.0, 27.0) | 25.0 (23.0, 27.0) | 25.0 (22.0, 27.0) | 0.008 |
| **Chloride, mEq/L, Median (IQR)** | 102.0 (99.0, 105.0) | 102.0 (99.0, 105.0) | 102.0 (99.0, 105.0) | 0.088 |
| **Creatinine, mg/dL, Median (IQR)** | 0.90 (0.70, 1.20) | 0.90 (0.70, 1.10) | 1.20 (0.90, 1.70) | <0.001 |
| **FBG, mg/dL, Median (IQR)** | 116 (98, 148) | 115 (98, 145) | 120 (100, 162) | <0.001 |
| **Hematocrit, %, Median (IQR)** | 36.9 (32.9, 40.4) | 37.1 (33.3, 40.7) | 35.5 (31.5, 39.5) | <0.001 |
| **Hemoglobin, g/dL, Median (IQR)** | 12.30 (10.80, 13.60) | 12.40 (11.00, 13.70) | 11.80 (10.30, 13.20) | <0.001 |
| **MCHC, g/dL, Median (IQR)** | 33.30 (32.40, 34.30) | 33.40 (32.40, 34.30) | 33.20 (32.20, 34.20) | <0.001 |
| **MCH, pg, Median (IQR)** | 30.00 (28.50, 31.50) | 30.00 (28.50, 31.50) | 30.00 (28.30, 31.50) | 0.2 |
| **MCV, fL, Median (IQR)** | 90 (86, 94) | 90 (86, 94) | 90 (86, 94) | <0.001 |
| **Platelet Count, K/uL, Median (IQR)** | 237 (183, 301) | 238 (185, 301) | 230 (175, 298) | <0.001 |
| **Potassium, mEq/L, Median (IQR)** | 4.10 (3.80, 4.50) | 4.10 (3.80, 4.50) | 4.30 (3.90, 4.70) | <0.001 |
| **RDW, %, Median (IQR)** | 13.80 (13.10, 14.90) | 13.70 (13.10, 14.70) | 14.20 (13.40, 15.40) | <0.001 |
| **RBC, m/uL, Median (IQR)** | 4.13 (3.65, 4.56) | 4.16 (3.69, 4.59) | 3.97 (3.47, 4.44) | <0.001 |
| **Sodium, mEq/L, Median (IQR)** | 138.0 (136.0, 141.0) | 138.0 (136.0, 141.0) | 138.0 (136.0, 141.0) | 0.064 |
| **WBC, K/uL, Median (IQR)** | 8.6 (6.5, 11.4) | 8.7 (6.5, 11.5) | 8.2 (6.2, 11.1) | <0.001 |
| **Medication history** |  |  |  |  |
| **Metformin, n (%)** | 2,297 (6.3%) | 1,772 (6.0%) | 525 (7.4%) | <0.001 |
| **Glimepiride, n (%)** | 71 (0.2%) | 51 (0.2%) | 20 (0.3%) | 0.062 |
| **Furosemide, n (%)** | 4,963 (14%) | 3,470 (12%) | 1,493 (21%) | <0.001 |
| **beta-Block, n(%)** | 6,862 (19%) | 4,810 (16%) | 2,052 (29%) | <0.001 |
| **Statin, n (%)** | 5,654 (15%) | 4,113 (14%) | 1,541 (22%) | <0.001 |
| **CCB, n (%)** | 6,724 (18%) | 4,823 (16%) | 1,901 (27%) | <0.001 |
| **ARB, n(%)** | 3,276 (9.0%) | 2,355 (8.0%) | 921 (13%) | <0.001 |
| **ACEI, n(%)** | 8,918 (24%) | 6,733 (23%) | 2,185 (31%) | <0.001 |

\# LOS, length of stay; DM, diabetes mellitus; MetS, metabolic syndrome; CKD, chronic kidney diseases; ePVS_admission, estimated plasma volume statute at admission; ePVS_discharge, estimated plasma volume statute at discharge; eGFR, estimated glomerular filtration rate; FBG, fast blood glucose; MCHC, mean corpuscular hemoglobin concentration; MCH, mean corpuscular hemoglobin; MCV, mean cell volume; RDW, red blood cell distribution width; RBC, red blood cell count; WBC, white blood cell count; CCB, calcium channel blockers; ARB, angiotensin II receptor blocker; ACEI, angiotensin-converting enzyme inhibitor.

# Table S5. Baseline characteristics of CKM2 patients who progress to CAD or non-CAD

| **Characteristic** | **Overall N = 36,505** | **Non-CAD N = 27,779** | **CAD N = 8,726** | ***P* value** |
| --- | --- | --- | --- | --- |
| **Demographic** |  |  |  |  |
| **Sex (Male), n (%)** | 16,388 (45%) | 12,117 (44%) | 4,271 (49%) | <0.001 |
| **Age, years** | 61 (50, 72) | 60 (48, 71) | 66 (56, 76) | <0.001 |
| **LOS, days** | 3.0 (1.0, 5.0) | 3.0 (1.0, 6.0) | 2.0 (1.0, 5.0) | <0.001 |
| **Medical history** |  |  |  |  |
| **DM, n (%)** | 11,780 (32%) | 8,356 (30%) | 3,424 (39%) | <0.001 |
| **Hypertension, n (%)** | 25,391 (70%) | 19,393 (70%) | 5,998 (69%) | 0.057 |
| **Hypertriglyceridemia, n (%)** | 1,670 (4.6%) | 1,471 (4.8%) | 199 (3.6%) | <0.001 |
| **MetS, n (%)** | 65 (0.2%) | 57 (0.2%) | 8 (<0.1%) | 0.028 |
| **CKD, n(%)** | 11,926 (33%) | 8,369 (30%) | 3,557 (41%) | <0.001 |
| **Anemia, n (%)** | 9,169 (25%) | 6,973 (25%) | 2,196 (25%) | >0.9 |
| **Hypothyroidism, n (%)** | 4,128 (11%) | 3,165 (11%) | 963 (11%) | 0.4 |
| **Hyperuricemia, n (%)** | 1,475 (4.0%) | 1,021 (3.7%) | 454 (5.2%) | <0.001 |
| **Lab result** |  |  |  |  |
| **ePVS_admission, (mL/g), Median (IQR)** | 5.14 (4.39, 6.18) | 5.12 (4.38, 6.16) | 5.19 (4.40, 6.23) | 0.006 |
| **ePVS_discharge, (mL/g), Median (IQR)** | 5.77 (4.82, 7.11) | 5.74 (4.80, 7.03) | 5.78 (4.83, 7.14) | 0.019 |
| **eGFR, mL/(min·1.73m^2), Median (IQR)** | 75 (54, 97) | 77 (56, 101) | 67 (48, 88) | <0.001 |
| **Anion Gap, mEq/L, Median (IQR)** | 15.0 (13.0, 17.0) | 14.0 (13.0, 17.0) | 15.0 (13.0, 17.0) | <0.001 |
| **Bicarbonate, mEq/L, Median (IQR)** | 25.0 (23.0, 27.0) | 25.0 (23.0, 27.0) | 25.0 (23.0, 28.0) | <0.001 |
| **Chloride, mEq/L, Median (IQR)** | 102.0 (99.0, 105.0) | 102.0 (99.0, 105.0) | 102.0 (99.0, 105.0) | 0.8 |
| **Creatinine, mg/dL, Median (IQR)** | 0.90 (0.70, 1.20) | 0.90 (0.70, 1.20) | 1.00 (0.80, 1.40) | <0.001 |
| **FBG, mg/dL, Median (IQR)** | 116 (98, 148) | 115 (98, 146) | 118 (99, 156) | <0.001 |
| **Hematocrit, %, Median (IQR)** | 36.9 (32.9, 40.4) | 36.9 (33.0, 40.5) | 36.7 (32.7, 40.3) | 0.005 |
| **Hemoglobin, g/dL, Median (IQR)** | 12.30 (10.80, 13.60) | 12.30 (10.90, 13.60) | 12.20 (10.80, 13.60) | 0.008 |
| **MCHC, g/dL, Median (IQR)** | 33.30 (32.40, 34.30) | 33.30 (32.40, 34.30) | 33.30 (32.30, 34.30) | 0.4 |
| **MCH, pg, Median (IQR)** | 30.00 (28.50, 31.50) | 30.10 (28.50, 31.50) | 30.00 (28.40, 31.50) | 0.027 |
| **MCV, fL, Median (IQR)** | 90 (86, 94) | 90 (86, 94) | 90 (86, 94) | 0.12 |
| **Platelet Count, K/uL, Median (IQR)** | 237 (183, 301) | 238 (183, 302) | 233 (182, 295) | <0.001 |
| **Potassium, mEq/L, Median (IQR)** | 4.10 (3.80, 4.50) | 4.10 (3.80, 4.50) | 4.20 (3.80, 4.60) | <0.001 |
| **RDW, %, Median (IQR)** | 13.80 (13.10, 14.90) | 13.80 (13.10, 14.80) | 13.90 (13.20, 15.00) | <0.001 |
| **RBC, m/uL, Median (IQR)** | 4.13 (3.65, 4.56) | 4.13 (3.65, 4.57) | 4.11 (3.63, 4.55) | 0.12 |
| **Sodium, mEq/L, Median (IQR)** | 138.0 (136.0, 141.0) | 138.0 (136.0, 141.0) | 138.0 (136.0, 141.0) | 0.3 |
| **WBC, K/uL, Median (IQR)** | 8.6 (6.5, 11.4) | 8.7 (6.5, 11.6) | 8.2 (6.3, 10.8) | <0.001 |
| **Medication history** |  |  |  |  |
| **Metformin, n (%)** | 2,297 (6.3%) | 1,587 (5.7%) | 710 (8.1%) | <0.001 |
| **Glimepiride, n (%)** | 71 (0.2%) | 48 (0.2%) | 23 (0.3%) | 0.093 |
| **Furosemide, n (%)** | 4,963 (14%) | 3,540 (13%) | 1,423 (16%) | <0.001 |
| **beta-Block, n(%)** | 6,862 (19%) | 4,418 (16%) | 2,444 (28%) | <0.001 |
| **Statin, n (%)** | 5,654 (15%) | 3,724 (13%) | 1,930 (22%) | <0.001 |
| **CCB, n (%)** | 6,724 (18%) | 4,695 (17%) | 2,029 (23%) | <0.001 |
| **ARB, n(%)** | 3,276 (9.0%) | 2,161 (7.8%) | 1,115 (13%) | <0.001 |
| **ACEI, n(%)** | 8,918 (24%) | 6,228 (22%) | 2,690 (31%) | <0.001 |

\# LOS, length of stay; DM, diabetes mellitus; MetS, metabolic syndrome; CKD, chronic kidney diseases; ePVS_admission, estimated plasma volume statute at admission; ePVS_discharge, estimated plasma volume statute at discharge; eGFR, estimated glomerular filtration rate; FBG, fast blood glucose; MCHC, mean corpuscular hemoglobin concentration; MCH, mean corpuscular hemoglobin; MCV, mean cell volume; RDW, red blood cell distribution width; RBC, red blood cell count; WBC, white blood cell count; CCB, calcium channel blockers; ARB, angiotensin II receptor blocker; ACEI, angiotensin-converting enzyme inhibitor.

# Table S6. Baseline characteristics of CKM2 patients who progress to HF or non-HF

| **Characteristic** | **Overall N = 36,505** | **Non-HF N = 30,921** | **HF N = 5,584** | ***P* value** |
| --- | --- | --- | --- | --- |
| **Demographic** |  |  |  |  |
| **Sex (Male), n (%)** | 16,388 (45%) | 13,847 (45%) | 2,541 (46%) | 0.3 |
| **Age, years** | 61 (50, 72) | 60 (48, 71) | 68 (58, 78) | <0.001 |
| **LOS, days** | 3.0 (1.0, 5.0) | 3.0 (1.0, 6.0) | 2.0 (1.0, 5.0) | <0.001 |
| **Medical history** |  |  |  |  |
| **DM, n (%)** | 11,780 (32%) | 9,437 (31%) | 2,343 (42%) | <0.001 |
| **Hypertension, n (%)** | 25,391 (70%) | 21,712 (70%) | 3,679 (66%) | <0.001 |
| **Hypertriglyceridemia, n (%)** | 1,670 (4.6%) | 1,349 (4.9%) | 321 (3.7%) | <0.001 |
| **MetS, n(%)** | 65 (0.2%) | 58 (0.2%) | 7 (0.1%) | 0.3 |
| **CKD, n (%)** | 11,926 (33%) | 9,266 (30%) | 2,660 (48%) | <0.001 |
| **Anemia, n(%)** | 9,169 (25%) | 7,638 (25%) | 1,531 (27%) | <0.001 |
| **Hypothyroidism, n (%)** | 4,128 (11%) | 3,412 (11%) | 716 (13%) | <0.001 |
| **Hyperuricemia, n (%)** | 1,475 (4.0%) | 1,123 (3.6%) | 352 (6.3%) | <0.001 |
| **Lab result** |  |  |  |  |
| **ePVS_admission, (mL/g), Median (IQR)** | 5.14 (4.39, 6.18) | 5.10 (4.36, 6.13) | 5.35 (4.52, 6.44) | <0.001 |
| **ePVS_discharge, (mL/g), Median (IQR)** | 5.77 (4.82, 7.11) | 5.74 (4.80, 7.08) | 5.95 (4.95, 7.26) | <0.001 |
| **eGFR, mL/(min·1.73m^2), Median (IQR)** | 75 (54, 97) | 77 (56, 100) | 61 (42, 83) | <0.001 |
| **Anion Gap, mEq/L, Median (IQR)** | 15.0 (13.0, 17.0) | 14.0 (13.0, 17.0) | 15.0 (13.0, 17.0) | <0.001 |
| **Bicarbonate, mEq/L, Median (IQR)** | 25.0 (23.0, 27.0) | 25.0 (23.0, 27.0) | 25.0 (23.0, 28.0) | <0.001 |
| **Chloride, mEq/L, Median (IQR)** | 102.0 (99.0, 105.0) | 102.0 (99.0, 105.0) | 102.0 (98.0, 105.0) | 0.004 |
| **Creatinine, mg/dL, Median (IQR)** | 0.90 (0.70, 1.20) | 0.90 (0.70, 1.20) | 1.10 (0.80, 1.50) | <0.001 |
| **FBG, mg/dL, Median (IQR)** | 116 (98, 148) | 115 (98, 146) | 119 (99, 158) | <0.001 |
| **Hematocrit, %, Median (IQR)** | 36.9 (32.9, 40.4) | 37.0 (33.0, 40.6) | 36.0 (32.0, 39.8) | <0.001 |
| **Hemoglobin, g/dL, Median (IQR)** | 12.30 (10.80, 13.60) | 12.30 (10.90, 13.60) | 12.00 (10.50, 13.30) | <0.001 |
| **MCHC, g/dL, Median (IQR)** | 33.30 (32.40, 34.30) | 33.40 (32.40, 34.30) | 33.20 (32.20, 34.20) | <0.001 |
| **MCH, pg, Median (IQR)** | 30.00 (28.50, 31.50) | 30.10 (28.50, 31.50) | 29.90 (28.30, 31.50) | 0.003 |
| **MCV, fL, Median (IQR)** | 90 (86, 94) | 90 (86, 94) | 90 (86, 94) | 0.15 |
| **Platelet Count, K/uL, Median (IQR)** | 237 (183, 301) | 237 (183, 301) | 233 (181, 297) | 0.023 |
| **Potassium, mEq/L, Median (IQR)** | 4.10 (3.80, 4.50) | 4.10 (3.80, 4.50) | 4.20 (3.90, 4.60) | <0.001 |
| **RDW, %, Median (IQR)** | 13.80 (13.10, 14.90) | 13.80 (13.10, 14.80) | 14.10 (13.30, 15.20) | <0.001 |
| **RBC, m/uL, Median (IQR)** | 4.13 (3.65, 4.56) | 4.14 (3.66, 4.57) | 4.04 (3.56, 4.49) | <0.001 |
| **Sodium, mEq/L, Median (IQR)** | 138.0 (136.0, 141.0) | 138.0 (136.0, 141.0) | 138.0 (136.0, 141.0) | 0.6 |
| **WBC, K/uL, Median (IQR)** | 8.6 (6.5, 11.4) | 8.6 (6.5, 11.5) | 8.3 (6.4, 11.0) | <0.001 |
| **Medication history** |  |  |  |  |
| **Metformin, n (%)** | 2,297 (6.3%) | 1,841 (6.0%) | 456 (8.2%) | <0.001 |
| **Glimepiride, n (%)** | 71 (0.2%) | 52 (0.2%) | 19 (0.3%) | 0.007 |
| **Furosemide, n (%)** | 4,963 (14%) | 3,760 (12%) | 1,203 (22%) | <0.001 |
| **beta-Block, n(%)** | 6,862 (19%) | 5,197 (17%) | 1,665 (30%) | <0.001 |
| **Statin, n (%)** | 5,654 (15%) | 4,452 (14%) | 1,202 (22%) | <0.001 |
| **CCB, n (%)** | 6,724 (18%) | 5,286 (17%) | 1,438 (26%) | <0.001 |
| **ARB, n(%)** | 3,276 (9.0%) | 2,467 (8.0%) | 809 (14%) | <0.001 |
| **ACEI, n(%)** | 8,918 (24%) | 7,117 (23%) | 1,801 (32%) | <0.001 |

\# LOS, length of stay; DM, diabetes mellitus; MetS, metabolic syndrome; CKD, chronic kidney diseases; ePVS_admission, estimated plasma volume statute at admission; ePVS_discharge, estimated plasma volume statute at discharge; eGFR, estimated glomerular filtration rate; FBG, fast blood glucose; MCHC, mean corpuscular hemoglobin concentration; MCH, mean corpuscular hemoglobin; MCV, mean cell volume; RDW, red blood cell distribution width; RBC, red blood cell count; WBC, white blood cell count; CCB, calcium channel blockers; ARB, angiotensin II receptor blocker; ACEI, angiotensin-converting enzyme inhibitor.

# Table S7. Baseline characteristics of CKM2 patients who progress to stroke or non-stroke

| **Characteristic** | **Overall N = 36,505** | **Non-Stroke N = 32,345** | **Stroke N = 4,160** | ***P* value** |
| --- | --- | --- | --- | --- |
| **Demographic** |  |  |  |  |
| **Sex (Male), n (%)** | 16,388 (45%) | 14,499 (45%) | 1,889 (45%) | 0.5 |
| **Age, years** | 61 (50, 72) | 61 (49, 71) | 67 (57, 77) | <0.001 |
| **LOS, days** | 3.0 (1.0, 5.0) | 3.0 (1.0, 5.0) | 2.0 (1.0, 5.0) | <0.001 |
| **Medical history** |  |  |  |  |
| **DM, n (%)** | 11,780 (32%) | 10,273 (32%) | 1,507 (36%) | <0.001 |
| **Hypertension, n (%)** | 25,391 (70%) | 22,466 (69%) | 2,925 (70%) | 0.3 |
| **Hypertriglyceridemia, n (%)** | 1,670 (4.6%) | 1,505 (4.7%) | 165 (4.0%) | 0.046 |
| **MetS, n (%)** | 65 (0.2%) | 59 (0.2%) | 6 (0.1%) | 0.6 |
| **CKD, n (%)** | 11,926 (33%) | 10,259 (32%) | 1,667 (40%) | <0.001 |
| **Anemia, n (%)** | 9,169 (25%) | 8,214 (25%) | 955 (23%) | <0.001 |
| **Hypothyroidism, n (%)** | 4,128 (11%) | 3,613 (11%) | 515 (12%) | 0.020 |
| **Hyperuricemia, n (%)** | 1,475 (4.0%) | 1,301 (4.0%) | 174 (4.2%) | 0.6 |
| **Lab result** |  |  |  |  |
| **ePVS_admission, (mL/g), Median (IQR)** | 5.14 (4.39, 6.18) | 5.13 (4.39, 6.18) | 5.17 (4.40, 6.19) | 0.4 |
| **ePVS_discharge, (mL/g), Median (IQR)** | 5.77 (4.82, 7.11) | 5.75 (4.81, 6.98) | 5.77 (4.82, 7.13) | 0.033 |
| **eGFR, mL/(min·1.73m^2), Median (IQR)** | 75 (54, 97) | 76 (55, 99) | 68 (50, 88) | <0.001 |
| **Anion Gap, mEq/L, Median (IQR)** | 15.0 (13.0, 17.0) | 15.0 (13.0, 17.0) | 15.0 (13.0, 17.0) | 0.13 |
| **Bicarbonate, mEq/L, Median (IQR)** | 25.0 (23.0, 27.0) | 25.0 (23.0, 27.0) | 25.0 (23.0, 28.0) | <0.001 |
| **Chloride, mEq/L, Median (IQR)** | 102.0 (99.0, 105.0) | 102.0 (99.0, 105.0) | 102.0 (99.0, 105.0) | <0.001 |
| **Creatinine, mg/dL, Median (IQR)** | 0.90 (0.70, 1.20) | 0.90 (0.70, 1.20) | 1.00 (0.80, 1.30) | <0.001 |
| **FBG, mg/dL, Median (IQR)** | 116 (98, 148) | 115 (98, 147) | 117 (99, 154) | <0.001 |
| **Hematocrit, %, Median (IQR)** | 36.9 (32.9, 40.4) | 36.9 (32.9, 40.4) | 36.7 (32.9, 40.4) | 0.3 |
| **Hemoglobin, g/dL, Median (IQR)** | 12.30 (10.80, 13.60) | 12.30 (10.80, 13.60) | 12.20 (10.80, 13.60) | 0.5 |
| **MCHC, g/dL, Median (IQR)** | 33.30 (32.40, 34.30) | 33.30 (32.40, 34.30) | 33.30 (32.30, 34.30) | 0.5 |
| **MCH, pg, Median (IQR)** | 30.00 (28.50, 31.50) | 30.00 (28.50, 31.50) | 30.20 (28.50, 31.60) | 0.013 |
| **MCV, fL, Median (IQR)** | 90 (86, 94) | 90 (86, 94) | 90 (86, 94) | 0.001 |
| **Platelet Count, K/uL, Median (IQR)** | 237 (183, 301) | 237 (183, 301) | 235 (185, 301) | 0.4 |
| **Potassium, mEq/L, Median (IQR)** | 4.10 (3.80, 4.50) | 4.10 (3.80, 4.50) | 4.10 (3.80, 4.50) | <0.001 |
| **RDW, %, Median (IQR)** | 13.80 (13.10, 14.90) | 13.80 (13.10, 14.90) | 13.80 (13.20, 14.90) | 0.019 |
| **RBC, m/uL, Median (IQR)** | 4.13 (3.65, 4.56) | 4.13 (3.65, 4.56) | 4.10 (3.63, 4.54) | 0.086 |
| **Sodium, mEq/L, Median (IQR)** | 138.0 (136.0, 141.0) | 138.0 (136.0, 141.0) | 139.0 (136.0, 141.0) | <0.001 |
| **WBC, K/uL, Median (IQR)** | 8.6 (6.5, 11.4) | 8.6 (6.5, 11.5) | 8.2 (6.4, 10.8) | <0.001 |
| **Medication history** |  |  |  |  |
| **Metformin, n (%)** | 2,297 (6.3%) | 2,003 (6.2%) | 294 (7.1%) | 0.029 |
| **Glimepiride, n (%)** | 71 (0.2%) | 62 (0.2%) | 9 (0.2%) | 0.7 |
| **Furosemide, n (%)** | 4,963 (14%) | 4,393 (14%) | 570 (14%) | 0.8 |
| **beta-Block, n(%)** | 6,862 (19%) | 5,719 (18%) | 1,143 (27%) | <0.001 |
| **Statin, n (%)** | 5,654 (15%) | 4,667 (14%) | 987 (24%) | <0.001 |
| **CCB, n (%)** | 6,724 (18%) | 5,693 (18%) | 1,031 (25%) | <0.001 |
| **ARB, n(%)** | 3,276 (9.0%) | 2,790 (8.6%) | 486 (12%) | <0.001 |
| **ACEI, n(%)** | 8,918 (24%) | 7,605 (24%) | 1,313 (32%) | <0.001 |

\# LOS, length of stay; DM, diabetes mellitus; MetS, metabolic syndrome; CKD, chronic kidney diseases; ePVS_admission, estimated plasma volume statute at admission; ePVS_discharge, estimated plasma volume statute at discharge; eGFR, estimated glomerular filtration rate; FBG, fast blood glucose; MCHC, mean corpuscular hemoglobin concentration; MCH, mean corpuscular hemoglobin; MCV, mean cell volume; RDW, red blood cell distribution width; RBC, red blood cell count; WBC, white blood cell count; CCB, calcium channel blockers; ARB, angiotensin II receptor blocker; ACEI, angiotensin-converting enzyme inhibitor.

**Table S8.** Mean absolute SHAP values for the six endpoints.

| **Characteristic** | **CKM4** | **CKM4a** | **CKM4b** | **CAD** | **HF** | **Stroke** |
| --- | --- | --- | --- | --- | --- | --- |
| **Age** | 957.6 | 696.4 | 303.0 | 407.5 | 333.4 | 162.8 |
| **ePVS_admission** | 197.0 | 63.6 | 119.4 | 92.8 | 98.2 | 26.0 |
| **Platelet Count** | 181.8 | 132.2 | 67.8 | 91.3 | 38.8 | 21.2 |
| **Sex** | 148.0 | 69.3 | 95.3 | 69.8 | 11.8 | 6.5 |
| **LOS** | 144.6 | 138.5 | 26.1 | 18.5 | 16.0 | 15.5 |
| **eGFR** | 133.3 | 138.7 | 270.3 | 17.1 | 91.6 | 23.8 |
| **ePVS_discharge** | 120.3 | 59.9 | 103.9 | 27.6 | 28.0 | 14.2 |
| **Bicarbonate** | 115.5 | 47.7 | 44.2 | 38.3 | 12.0 | 31.3 |
| **Potassium** | 112.7 | 32.1 | 88.4 | 89.4 | 80.2 | 12.2 |
| **CKD** | 40.1 | 26.6 | 120.5 | 64.2 | 44.0 | 16.2 |
| **WBC** | 38.7 | 32.0 | 22.4 | 24.1 | 11.6 | 14.9 |
| **Anemia** | 35.0 | 21.3 | 21.6 | 19.3 | 15.0 | 6.1 |
| **Hyperuricemia** | 34.7 | 22.1 | 20.2 | 20.8 | 10.1 | 6.6 |
| **Hypertriglyceridemia** | 29.2 | 26.7 | 25.0 | 22.8 | 13.5 | 11.7 |
| **Hypertension** | 28.4 | 61.1 | 44.2 | 62.3 | 11.0 | 6.7 |
| **DM** | 27.1 | 19.8 | 66.8 | 68.9 | 65.0 | 10.9 |
| **MetS** | 28.1 | 21.5 | 21.2 | 12.4 | 8.5 | 3.1 |
| **Chloride** | 20.5 | 13.5 | 12.5 | 7.3 | 10.5 | 4.2 |
| **Sodium** | 19.4 | 11.5 | 10.1 | 6.2 | 11.3 | 3.8 |
| **Metformin** | 24.2 | 18.4 | 17.5 | 9.1 | 6.3 | 4.8 |
| **Glimepiride** | 22.3 | 16.1 | 15.4 | 7.5 | 5.2 | 3.5 |
| **Furosemide** | 26.1 | 19.5 | 18.5 | 4.5 | 11.5 | 2.4 |
| **beta-Blocker** | 17.2 | 10.3 | 9.5 | 13.1 | 11.4 | 4.5 |
| **Statin** | 18.1 | 9.4 | 8.2 | 16.3 | 4.1 | 5.8 |
| **CCB** | 12.3 | 6.5 | 5.5 | 14.2 | 3.4 | 5.1 |
| **ARB** | 21.4 | 12.1 | 11.5 | 8.3 | 9.5 | 2.5 |
| **ACEI** | 23.1 | 14.3 | 13.5 | 10.2 | 10.4 | 1.8 |

\# LOS, length of stay; DM, diabetes mellitus; CKD, chronic kidney diseases; ePVS_admission, estimated plasma volume statute at admission; ePVS_discharge, estimated plasma volume statute at discharge; eGFR, estimated glomerular filtration rate; WBC, white blood cell count; CCB, calcium channel blockers; ARB, angiotensin II receptor blocker; ACEI, angiotensin-converting enzyme inhibitor.

**Table S9.** OOB prediction error and discriminative performance of the random forest models used for SHAP analysis across various endpoints.

| **Endpoint** | **C-index** | **Brier 1-year** | **Brier 3-year** | **Brier 5-year** |
| --- | --- | --- | --- | --- |
| **CKM4** | 0.6504 | 0.1493 | 0.1806 | 0.2011 |
| **CKM4a** | 0.6386 | 0.0036 | 0.0038 | 0.0041 |
| **CKM4b** | 0.7287 | 0.0034 | 0.0043 | 0.0047 |
| **CAD** | 0.6021 | 0.0925 | 0.1155 | 0.1316 |
| **HF** | 0.6308 | 0.0099 | 0.0115 | 0.0128 |
| **Stroke** | 0.6970 | 0.0713 | 0.0788 | 0.0839 |

# **Table S10.** The Cox analysis for ePVS quartiles in various endpoints

|  |  |  | **ePVS_admission** | | **ePVS_discharge** | |
| --- | --- | --- | --- | --- | --- | --- |
| **Endpoints** | **Characteristic** | **Model** | **HR (95% CI)** | ***P* value** | **HR (95% CI)** | ***P* value** |
| **CKM4** | ePVS | Model 0 |  |  |  |  |
| **CKM4** | Q1 | Model 0 | ref | ref | ref | ref |
| **CKM4** | Q2 | Model 0 | 1.00 (0.96, 1.05) | >0.9 | 1.07 (1.02, 1.12) | 0.005 |
| **CKM4** | Q3 | Model 0 | 1.17 (1.11, 1.22) | <0.001 | 1.23 (1.18, 1.29) | <0.001 |
| **CKM4** | Q4 | Model 0 | 1.46 (1.40, 1.53) | <0.001 | 1.50 (1.43, 1.56) | <0.001 |
| **CKM4** | ePVS | Model 1 |  |  |  |  |
| **CKM4** | Q1 | Model 1 | ref | ref | ref | ref |
| **CKM4** | Q2 | Model 1 | 1.04 (0.99, 1.09) | 0.13 | 1.08 (1.04, 1.13) | <0.001 |
| **CKM4** | Q3 | Model 1 | 1.21 (1.16, 1.27) | <0.001 | 1.25 (1.19, 1.30) | <0.001 |
| **CKM4** | Q4 | Model 1 | 1.49 (1.43, 1.56) | <0.001 | 1.47 (1.41, 1.54) | <0.001 |
| **CKM4** | ePVS | Model 2 |  |  |  |  |
| **CKM4** | Q1 | Model 2 | ref | ref | ref | ref |
| **CKM4** | Q2 | Model 2 | 1.03 (0.98, 1.07) | 0.3 | 1.07 (1.02, 1.12) | 0.007 |
| **CKM4** | Q3 | Model 2 | 1.18 (1.12, 1.24) | <0.001 | 1.20 (1.14, 1.26) | <0.001 |
| **CKM4** | Q4 | Model 2 | 1.42 (1.35, 1.49) | <0.001 | 1.39 (1.32, 1.47) | <0.001 |
| **CKM4** | ePVS | Model 3 |  |  |  |  |
| **CKM4** | Q1 | Model 3 | ref | ref | ref | ref |
| **CKM4** | Q2 | Model 3 | 1.04 (0.99, 1.09) | 0.12 | 1.06 (1.01, 1.11) | 0.010 |
| **CKM4** | Q3 | Model 3 | 1.18 (1.13, 1.24) | <0.001 | 1.18 (1.13, 1.24) | <0.001 |
| **CKM4** | Q4 | Model 3 | 1.41 (1.34, 1.49) | <0.001 | 1.35 (1.28, 1.42) | <0.001 |
| **CKM4** | ePVS | Model 4 |  |  |  |  |
| **CKM4** | Q1 | Model 4 | ref | ref | ref | ref |
| **CKM4** | Q2 | Model 4 | 1.04 (0.99, 1.09) | 0.11 | 1.06 (1.02, 1.11) | 0.008 |
| **CKM4** | Q3 | Model 4 | 1.19 (1.13, 1.25) | <0.001 | 1.17 (1.12, 1.23) | <0.001 |
| **CKM4** | Q4 | Model 4 | 1.43 (1.36, 1.50) | <0.001 | 1.35 (1.28, 1.42) | <0.001 |
| **CKM4a** | ePVS | Model 0 |  |  |  |  |
| **CKM4a** | Q1 | Model 0 | ref | ref | ref | ref |
| **CKM4a** | Q2 | Model 0 | 0.98 (0.93, 1.02) | 0.3 | 1.03 (0.98, 1.08) | 0.3 |
| **CKM4a** | Q3 | Model 0 | 1.08 (1.03, 1.14) | 0.002 | 1.09 (1.04, 1.15) | <0.001 |
| **CKM4a** | Q4 | Model 0 | 1.16 (1.10, 1.22) | <0.001 | 1.17 (1.11, 1.23) | <0.001 |
| **CKM4a** | ePVS | Model 1 |  |  |  |  |
| **CKM4a** | Q1 | Model 1 | ref | ref | ref | ref |
| **CKM4a** | Q2 | Model 1 | 0.99 (0.94, 1.04) | 0.8 | 1.03 (0.98, 1.08) | 0.3 |
| **CKM4a** | Q3 | Model 1 | 1.10 (1.05, 1.16) | <0.001 | 1.08 (1.03, 1.14) | 0.002 |
| **CKM4a** | Q4 | Model 1 | 1.17 (1.11, 1.23) | <0.001 | 1.13 (1.08, 1.20) | <0.001 |
| **CKM4a** | ePVS | Model 2 |  |  |  |  |
| **CKM4a** | Q1 | Model 2 | ref | ref | ref | ref |
| **CKM4a** | Q2 | Model 2 | 1.00 (0.95, 1.05) | 0.9 | 1.04 (0.99, 1.10) | 0.11 |
| **CKM4a** | Q3 | Model 2 | 1.12 (1.06, 1.18) | <0.001 | 1.11 (1.06, 1.17) | <0.001 |
| **CKM4a** | Q4 | Model 2 | 1.21 (1.14, 1.28) | <0.001 | 1.18 (1.11, 1.25) | <0.001 |
| **CKM4a** | ePVS | Model 3 |  |  |  |  |
| **CKM4a** | Q1 | Model 3 | ref | ref | ref | ref |
| **CKM4a** | Q2 | Model 3 | 0.99 (0.95, 1.05) | 0.8 | 1.04 (0.99, 1.09) | 0.2 |
| **CKM4a** | Q3 | Model 3 | 1.11 (1.05, 1.17) | <0.001 | 1.10 (1.04, 1.16) | <0.001 |
| **CKM4a** | Q4 | Model 3 | 1.19 (1.12, 1.26) | <0.001 | 1.15 (1.09, 1.22) | <0.001 |
| **CKM4a** | ePVS | Model 4 |  |  |  |  |
| **CKM4a** | Q1 | Model 4 | ref | ref | ref | ref |
| **CKM4a** | Q2 | Model 4 | 1.00 (0.95, 1.05) | >0.9 | 1.04 (0.99, 1.09) | 0.11 |
| **CKM4a** | Q3 | Model 4 | 1.13 (1.07, 1.19) | <0.001 | 1.10 (1.04, 1.16) | <0.001 |
| **CKM4a** | Q4 | Model 4 | 1.22 (1.15, 1.29) | <0.001 | 1.17 (1.10, 1.24) | <0.001 |
| **CKM4b** | ePVS | Model 0 |  |  |  |  |
| **CKM4b** | Q1 | Model 0 | ref | ref | ref | ref |
| **CKM4b** | Q2 | Model 0 | 1.09 (1.01, 1.17) | 0.023 | 1.26 (1.17, 1.35) | <0.001 |
| **CKM4b** | Q3 | Model 0 | 1.46 (1.37, 1.57) | <0.001 | 1.73 (1.61, 1.85) | <0.001 |
| **CKM4b** | Q4 | Model 0 | 2.27 (2.12, 2.42) | <0.001 | 2.36 (2.21, 2.53) | <0.001 |
| **CKM4b** | ePVS | Model 1 |  |  |  |  |
| **CKM4b** | Q1 | Model 1 | ref | ref | ref | ref |
| **CKM4b** | Q2 | Model 1 | 1.18 (1.10, 1.28) | <0.001 | 1.34 (1.24, 1.44) | <0.001 |
| **CKM4b** | Q3 | Model 1 | 1.62 (1.51, 1.74) | <0.001 | 1.85 (1.72, 1.98) | <0.001 |
| **CKM4b** | Q4 | Model 1 | 2.47 (2.31, 2.65) | <0.001 | 2.46 (2.30, 2.64) | <0.001 |
| **CKM4b** | ePVS | Model 2 |  |  |  |  |
| **CKM4b** | Q1 | Model 2 | ref | ref | ref | ref |
| **CKM4b** | Q2 | Model 2 | 1.13 (1.05, 1.21) | 0.002 | 1.22 (1.13, 1.31) | <0.001 |
| **CKM4b** | Q3 | Model 2 | 1.40 (1.31, 1.51) | <0.001 | 1.52 (1.41, 1.64) | <0.001 |
| **CKM4b** | Q4 | Model 2 | 1.99 (1.85, 2.14) | <0.001 | 1.94 (1.79, 2.09) | <0.001 |
| **CKM4b** | ePVS | Model 3 |  |  |  |  |
| **CKM4b** | Q1 | Model 3 | ref | ref | ref | ref |
| **CKM4b** | Q2 | Model 3 | 1.14 (1.06, 1.23) | <0.001 | 1.19 (1.11, 1.29) | <0.001 |
| **CKM4b** | Q3 | Model 3 | 1.39 (1.29, 1.50) | <0.001 | 1.45 (1.35, 1.56) | <0.001 |
| **CKM4b** | Q4 | Model 3 | 1.90 (1.76, 2.05) | <0.001 | 1.76 (1.63, 1.91) | <0.001 |
| **CKM4b** | ePVS | Model 4 |  |  |  |  |
| **CKM4b** | Q1 | Model 4 | ref | ref | ref | ref |
| **CKM4b** | Q2 | Model 4 | 1.13 (1.05, 1.22) | <0.001 | 1.19 (1.10, 1.28) | <0.001 |
| **CKM4b** | Q3 | Model 4 | 1.38 (1.28, 1.48) | <0.001 | 1.42 (1.32, 1.53) | <0.001 |
| **CKM4b** | Q4 | Model 4 | 1.88 (1.74, 2.03) | <0.001 | 1.73 (1.60, 1.88) | <0.001 |
| **CAD** | ePVS | Model 0 |  |  |  |  |
| **CAD** | Q1 | Model 0 | ref | ref | ref | ref |
| **CAD** | Q2 | Model 0 | 0.97 (0.92, 1.03) | 0.4 | 1.04 (0.98, 1.10) | 0.2 |
| **CAD** | Q3 | Model 0 | 1.16 (1.09, 1.23) | <0.001 | 1.15 (1.09, 1.22) | <0.001 |
| **CAD** | Q4 | Model 0 | 1.41 (1.33, 1.49) | <0.001 | 1.32 (1.24, 1.40) | <0.001 |
| **CAD** | ePVS | Model 1 |  |  |  |  |
| **CAD** | Q1 | Model 1 | ref | ref | ref | ref |
| **CAD** | Q2 | Model 1 | 1.02 (0.96, 1.08) | 0.5 | 1.07 (1.01, 1.14) | 0.023 |
| **CAD** | Q3 | Model 1 | 1.23 (1.16, 1.31) | <0.001 | 1.20 (1.13, 1.27) | <0.001 |
| **CAD** | Q4 | Model 1 | 1.50 (1.41, 1.59) | <0.001 | 1.37 (1.28, 1.46) | <0.001 |
| **CAD** | ePVS | Model 2 |  |  |  |  |
| **CAD** | Q1 | Model 2 | ref | ref | ref | ref |
| **CAD** | Q2 | Model 2 | 1.00 (0.94, 1.06) | >0.9 | 1.04 (0.98, 1.10) | 0.2 |
| **CAD** | Q3 | Model 2 | 1.18 (1.11, 1.25) | <0.001 | 1.12 (1.05, 1.19) | <0.001 |
| **CAD** | Q4 | Model 2 | 1.40 (1.31, 1.49) | <0.001 | 1.25 (1.16, 1.33) | <0.001 |
| **CAD** | ePVS | Model 3 |  |  |  |  |
| **CAD** | Q1 | Model 3 | ref | ref | ref | ref |
| **CAD** | Q2 | Model 3 | 1.01 (0.95, 1.08) | 0.7 | 1.03 (0.97, 1.10) | 0.3 |
| **CAD** | Q3 | Model 3 | 1.18 (1.11, 1.26) | <0.001 | 1.10 (1.04, 1.18) | 0.002 |
| **CAD** | Q4 | Model 3 | 1.39 (1.30, 1.49) | <0.001 | 1.20 (1.12, 1.29) | <0.001 |
| **CAD** | ePVS | Model 4 |  |  |  |  |
| **CAD** | Q1 | Model 4 | ref | ref | ref | ref |
| **CAD** | Q2 | Model 4 | 1.01 (0.95, 1.07) | 0.8 | 1.04 (0.98, 1.11) | 0.2 |
| **CAD** | Q3 | Model 4 | 1.18 (1.11, 1.26) | <0.001 | 1.10 (1.03, 1.17) | 0.003 |
| **CAD** | Q4 | Model 4 | 1.40 (1.30, 1.50) | <0.001 | 1.22 (1.13, 1.31) | <0.001 |
| **HF** | ePVS | Model 0 |  |  |  |  |
| **HF** | Q1 | Model 0 | ref | ref | ref | ref |
| **HF** | Q2 | Model 0 | 1.10 (1.01, 1.19) | 0.023 | 1.18 (1.09, 1.27) | <0.001 |
| **HF** | Q3 | Model 0 | 1.39 (1.29, 1.50) | <0.001 | 1.45 (1.34, 1.56) | <0.001 |
| **HF** | Q4 | Model 0 | 1.90 (1.76, 2.04) | <0.001 | 1.80 (1.67, 1.95) | <0.001 |
| **HF** | ePVS | Model 1 |  |  |  |  |
| **HF** | Q1 | Model 1 | ref | ref | ref | ref |
| **HF** | Q2 | Model 1 | 1.12 (1.03, 1.21) | 0.006 | 1.18 (1.09, 1.27) | <0.001 |
| **HF** | Q3 | Model 1 | 1.42 (1.32, 1.54) | <0.001 | 1.44 (1.34, 1.56) | <0.001 |
| **HF** | Q4 | Model 1 | 1.93 (1.79, 2.08) | <0.001 | 1.79 (1.66, 1.94) | <0.001 |
| **HF** | ePVS | Model 2 |  |  |  |  |
| **HF** | Q1 | Model 2 | ref | ref | ref | ref |
| **HF** | Q2 | Model 2 | 1.09 (1.00, 1.18) | 0.042 | 1.11 (1.03, 1.20) | 0.009 |
| **HF** | Q3 | Model 2 | 1.31 (1.21, 1.42) | <0.001 | 1.28 (1.18, 1.39) | <0.001 |
| **HF** | Q4 | Model 2 | 1.71 (1.57, 1.86) | <0.001 | 1.54 (1.42, 1.68) | <0.001 |
| **HF** | ePVS | Model 3 |  |  |  |  |
| **HF** | Q1 | Model 3 | ref | ref | ref | ref |
| **HF** | Q2 | Model 3 | 1.12 (1.03, 1.21) | 0.006 | 1.11 (1.03, 1.20) | 0.008 |
| **HF** | Q3 | Model 3 | 1.36 (1.25, 1.48) | <0.001 | 1.28 (1.18, 1.39) | <0.001 |
| **HF** | Q4 | Model 3 | 1.80 (1.65, 1.96) | <0.001 | 1.53 (1.40, 1.67) | <0.001 |
| **HF** | ePVS | Model 4 |  |  |  |  |
| **HF** | Q1 | Model 4 | ref | ref | ref | ref |
| **HF** | Q2 | Model 4 | 1.11 (1.02, 1.20) | 0.012 | 1.11 (1.03, 1.20) | 0.009 |
| **HF** | Q3 | Model 4 | 1.35 (1.24, 1.46) | <0.001 | 1.26 (1.16, 1.36) | <0.001 |
| **HF** | Q4 | Model 4 | 1.76 (1.61, 1.92) | <0.001 | 1.50 (1.37, 1.64) | <0.001 |
| **Stroke** | ePVS | Model 0 |  |  |  |  |
| **Stroke** | Q1 | Model 0 | ref | ref | ref | ref |
| **Stroke** | Q2 | Model 0 | 0.96 (0.88, 1.05) | 0.4 | 1.05 (0.97, 1.15) | 0.2 |
| **Stroke** | Q3 | Model 0 | 1.15 (1.06, 1.26) | <0.001 | 1.20 (1.10, 1.30) | <0.001 |
| **Stroke** | Q4 | Model 0 | 1.34 (1.23, 1.46) | <0.001 | 1.27 (1.16, 1.39) | <0.001 |
| **Stroke** | ePVS | Model 1 |  |  |  |  |
| **Stroke** | Q1 | Model 1 | ref | ref | ref | ref |
| **Stroke** | Q2 | Model 1 | 0.97 (0.89, 1.06) | 0.5 | 1.04 (0.95, 1.13) | 0.4 |
| **Stroke** | Q3 | Model 1 | 1.17 (1.07, 1.27) | <0.001 | 1.18 (1.08, 1.28) | <0.001 |
| **Stroke** | Q4 | Model 1 | 1.33 (1.22, 1.46) | <0.001 | 1.23 (1.12, 1.35) | <0.001 |
| **Stroke** | ePVS | Model 2 |  |  |  |  |
| **Stroke** | Q1 | Model 2 | ref | ref | ref | ref |
| **Stroke** | Q2 | Model 2 | 0.97 (0.89, 1.06) | 0.5 | 1.03 (0.94, 1.12) | 0.5 |
| **Stroke** | Q3 | Model 2 | 1.16 (1.07, 1.27) | <0.001 | 1.17 (1.07, 1.28) | <0.001 |
| **Stroke** | Q4 | Model 2 | 1.36 (1.23, 1.49) | <0.001 | 1.23 (1.12, 1.36) | <0.001 |
| **Stroke** | ePVS | Model 3 |  |  |  |  |
| **Stroke** | Q1 | Model 3 | ref | ref | ref | ref |
| **Stroke** | Q2 | Model 3 | 0.98 (0.90, 1.07) | 0.7 | 1.02 (0.94, 1.12) | 0.6 |
| **Stroke** | Q3 | Model 3 | 1.16 (1.06, 1.27) | 0.001 | 1.14 (1.04, 1.24) | 0.005 |
| **Stroke** | Q4 | Model 3 | 1.34 (1.21, 1.48) | <0.001 | 1.17 (1.06, 1.29) | 0.003 |
| **Stroke** | ePVS | Model 4 |  |  |  |  |
| **Stroke** | Q1 | Model 4 | ref | ref | ref | ref |
| **Stroke** | Q2 | Model 4 | 0.99 (0.90, 1.08) | 0.8 | 1.03 (0.94, 1.12) | 0.5 |
| **Stroke** | Q3 | Model 4 | 1.16 (1.06, 1.27) | 0.001 | 1.13 (1.03, 1.24) | 0.008 |
| **Stroke** | Q4 | Model 4 | 1.38 (1.25, 1.52) | <0.001 | 1.21 (1.09, 1.34) | <0.001 |

#Model 0: unadjusted; Model 1: adjusted with sex, age, and LOS; Model 2: adjusted with Model 1 and DM, hypertension, hypertriglyceridemia, MetS, CKD, anemia, hyperuricemia; Model 3: adjusted with Model 2 and bicarbonate, chloride, eGFR, platelet count, potassium, sodium, and WBC; Model 4: adjusted with Model 3 and metformin, glimepiride, furosemide, beta-block, statin, CCB, ARB, and ACEI.

# Table S11. The interaction of age and sex influences the ePVS quartiles result of Model 4

|  |  |  | **ePVS_admission** | | | **ePVS_discharge** | | |
| --- | --- | --- | --- | --- | --- | --- | --- | --- |
| **Endpoints** | **Quartiles** | **Subgroup** | **HR (95% CI)** | ***P* value** | ***P* for interaction** | **HR (95% CI)** | ***P* value** | ***P* for interaction** |
| **CKM4** |  | Sex |  |  | 0.094 |  |  | 0.625 |
| **CKM4** | ePVS | F |  |  |  |  |  |  |
| **CKM4** | Q1 | F | ref | ref |  | ref | ref |  |
| **CKM4** | Q2 | F | 0.98 (0.91, 1.05) | 0.5 |  | 1.03 (0.97, 1.11) | 0.3 |  |
| **CKM4** | Q3 | F | 1.14 (1.07, 1.23) | <0.001 |  | 1.15 (1.08, 1.24) | <0.001 |  |
| **CKM4** | Q4 | F | 1.35 (1.25, 1.45) | <0.001 |  | 1.31 (1.22, 1.42) | <0.001 |  |
| **CKM4** | ePVS | M |  |  |  |  |  |  |
| **CKM4** | Q1 | M | ref | ref |  | ref | ref |  |
| **CKM4** | Q2 | M | 1.10 (1.03, 1.17) | 0.003 |  | 1.11 (1.04, 1.18) | 0.001 |  |
| **CKM4** | Q3 | M | 1.24 (1.15, 1.32) | <0.001 |  | 1.22 (1.14, 1.30) | <0.001 |  |
| **CKM4** | Q4 | M | 1.53 (1.42, 1.64) | <0.001 |  | 1.42 (1.31, 1.52) | <0.001 |  |
| **CKM4** |  | Age |  |  | 0.03 |  |  | 0.009 |
| **CKM4** | ePVS | > 60 |  |  |  |  |  |  |
| **CKM4** | Q1 | > 60 | ref | ref |  | ref | ref |  |
| **CKM4** | Q2 | > 60 | 1.08 (1.02, 1.15) | 0.011 |  | 1.10 (1.04, 1.17) | <0.001 |  |
| **CKM4** | Q3 | > 60 | 1.19 (1.12, 1.27) | <0.001 |  | 1.20 (1.13, 1.28) | <0.001 |  |
| **CKM4** | Q4 | > 60 | 1.44 (1.35, 1.54) | <0.001 |  | 1.37 (1.28, 1.46) | <0.001 |  |
| **CKM4** | ePVS | ≤ 60 |  |  |  |  |  |  |
| **CKM4** | Q1 | ≤ 60 | ref | ref |  | ref | ref |  |
| **CKM4** | Q2 | ≤ 60 | 0.98 (0.91, 1.06) | 0.7 |  | 1.01 (0.93, 1.09) | 0.8 |  |
| **CKM4** | Q3 | ≤ 60 | 1.20 (1.11, 1.30) | <0.001 |  | 1.13 (1.04, 1.22) | 0.002 |  |
| **CKM4** | Q4 | ≤ 60 | 1.40 (1.29, 1.53) | <0.001 |  | 1.31 (1.20, 1.43) | <0.001 |  |
| **CKM4a** |  | Sex |  |  | 0.066 |  |  | 0.272 |
| **CKM4a** | ePVS | F |  |  |  |  |  |  |
| **CKM4a** | Q1 | F | ref | ref |  | ref | ref |  |
| **CKM4a** | Q2 | F | 0.94 (0.87, 1.01) | 0.11 |  | 1.02 (0.94, 1.09) | 0.7 |  |
| **CKM4a** | Q3 | F | 1.09 (1.01, 1.18) | 0.020 |  | 1.10 (1.02, 1.19) | 0.010 |  |
| **CKM4a** | Q4 | F | 1.19 (1.10, 1.29) | <0.001 |  | 1.16 (1.07, 1.27) | <0.001 |  |
| **CKM4a** | ePVS | M |  |  |  |  |  |  |
| **CKM4a** | Q1 | M | ref | ref |  | ref | ref |  |
| **CKM4a** | Q2 | M | 1.07 (1.00, 1.14) | 0.063 |  | 1.10 (1.02, 1.18) | 0.010 |  |
| **CKM4a** | Q3 | M | 1.16 (1.08, 1.26) | <0.001 |  | 1.11 (1.03, 1.20) | 0.010 |  |
| **CKM4a** | Q4 | M | 1.25 (1.14, 1.36) | <0.001 |  | 1.19 (1.09, 1.30) | <0.001 |  |
| **CKM4a** |  | Age |  |  | 0.045 |  |  | 0.012 |
| **CKM4a** | ePVS | > 60 |  |  |  |  |  |  |
| **CKM4a** | Q1 | > 60 | ref | ref |  | ref | ref |  |
| **CKM4a** | Q2 | > 60 | 1.04 (0.97, 1.11) | 0.3 |  | 1.08 (1.01, 1.15) | 0.016 |  |
| **CKM4a** | Q3 | > 60 | 1.14 (1.07, 1.22) | <0.001 |  | 1.13 (1.06, 1.21) | <0.001 |  |
| **CKM4a** | Q4 | > 60 | 1.25 (1.16, 1.34) | <0.001 |  | 1.19 (1.10, 1.28) | <0.001 |  |
| **CKM4a** | ePVS | ≤ 60 |  |  |  |  |  |  |
| **CKM4a** | Q1 | ≤ 60 | ref | ref |  | ref | ref |  |
| **CKM4a** | Q2 | ≤ 60 | 0.96 (0.88, 1.04) | 0.3 |  | 0.99 (0.91, 1.07) | 0.7 |  |
| **CKM4a** | Q3 | ≤ 60 | 1.13 (1.03, 1.23) | 0.008 |  | 1.06 (0.97, 1.16) | 0.2 |  |
| **CKM4a** | Q4 | ≤ 60 | 1.18 (1.07, 1.30) | 0.001 |  | 1.12 (1.02, 1.24) | 0.023 |  |
| **CKM4b** |  | Sex |  |  | 0.002 |  |  | 0.041 |
| **CKM4b** | ePVS | F |  |  |  |  |  |  |
| **CKM4b** | Q1 | F | ref | ref |  | ref | ref |  |
| **CKM4b** | Q2 | F | 0.95 (0.85, 1.07) | 0.4 |  | 1.09 (0.97, 1.22) | 0.2 |  |
| **CKM4b** | Q3 | F | 1.22 (1.09, 1.37) | <0.001 |  | 1.36 (1.21, 1.53) | <0.001 |  |
| **CKM4b** | Q4 | F | 1.58 (1.41, 1.79) | <0.001 |  | 1.59 (1.40, 1.80) | <0.001 |  |
| **CKM4b** | ePVS | M |  |  |  |  |  |  |
| **CKM4b** | Q1 | M | ref | ref |  | ref | ref |  |
| **CKM4b** | Q2 | M | 1.31 (1.19, 1.44) | <0.001 |  | 1.32 (1.20, 1.46) | <0.001 |  |
| **CKM4b** | Q3 | M | 1.52 (1.37, 1.68) | <0.001 |  | 1.52 (1.38, 1.68) | <0.001 |  |
| **CKM4b** | Q4 | M | 2.17 (1.95, 2.41) | <0.001 |  | 1.91 (1.72, 2.13) | <0.001 |  |
| **CKM4b** |  | Age |  |  | 0.581 |  |  | 0.723 |
| **CKM4b** | ePVS | > 60 |  |  |  |  |  |  |
| **CKM4b** | Q1 | > 60 | ref | ref |  | ref | ref |  |
| **CKM4b** | Q2 | > 60 | 1.15 (1.04, 1.26) | 0.004 |  | 1.20 (1.09, 1.31) | <0.001 |  |
| **CKM4b** | Q3 | > 60 | 1.33 (1.21, 1.45) | <0.001 |  | 1.40 (1.27, 1.53) | <0.001 |  |
| **CKM4b** | Q4 | > 60 | 1.81 (1.64, 1.99) | <0.001 |  | 1.65 (1.49, 1.83) | <0.001 |  |
| **CKM4b** | ePVS | ≤ 60 |  |  |  |  |  |  |
| **CKM4b** | Q1 | ≤ 60 | ref | ref |  | ref | ref |  |
| **CKM4b** | Q2 | ≤ 60 | 1.11 (0.98, 1.26) | 0.10 |  | 1.16 (1.02, 1.31) | 0.025 |  |
| **CKM4b** | Q3 | ≤ 60 | 1.47 (1.30, 1.67) | <0.001 |  | 1.45 (1.28, 1.64) | <0.001 |  |
| **CKM4b** | Q4 | ≤ 60 | 2.03 (1.78, 2.31) | <0.001 |  | 1.86 (1.62, 2.13) | <0.001 |  |
| **CAD** |  | Sex |  |  | 0.01 |  |  | 0.139 |
| **CAD** | ePVS | F |  |  |  |  |  |  |
| **CAD** | Q1 | F | ref | ref |  | ref | ref |  |
| **CAD** | Q2 | F | 0.91 (0.82, 1.00) | 0.039 |  | 0.96 (0.88, 1.05) | 0.4 |  |
| **CAD** | Q3 | F | 1.10 (1.00, 1.20) | 0.051 |  | 1.03 (0.94, 1.13) | 0.5 |  |
| **CAD** | Q4 | F | 1.25 (1.13, 1.38) | <0.001 |  | 1.12 (1.01, 1.25) | 0.027 |  |
| **CAD** | ePVS | M |  |  |  |  |  |  |
| **CAD** | Q1 | M | ref | ref |  | ref | ref |  |
| **CAD** | Q2 | M | 1.10 (1.01, 1.19) | 0.028 |  | 1.12 (1.03, 1.22) | 0.006 |  |
| **CAD** | Q3 | M | 1.24 (1.14, 1.36) | <0.001 |  | 1.17 (1.07, 1.28) | <0.001 |  |
| **CAD** | Q4 | M | 1.55 (1.40, 1.71) | <0.001 |  | 1.32 (1.19, 1.46) | <0.001 |  |
| **CAD** |  | Age |  |  | 0.08 |  |  | 0.003 |
| **CAD** | ePVS | > 60 |  |  |  |  |  |  |
| **CAD** | Q1 | > 60 | ref | ref |  | ref | ref |  |
| **CAD** | Q2 | > 60 | 1.05 (0.97, 1.13) | 0.2 |  | 1.10 (1.02, 1.19) | 0.013 |  |
| **CAD** | Q3 | > 60 | 1.21 (1.12, 1.31) | <0.001 |  | 1.16 (1.07, 1.25) | <0.001 |  |
| **CAD** | Q4 | > 60 | 1.45 (1.32, 1.58) | <0.001 |  | 1.28 (1.17, 1.40) | <0.001 |  |
| **CAD** | ePVS | ≤ 60 |  |  |  |  |  |  |
| **CAD** | Q1 | ≤ 60 | ref | ref |  | ref | ref |  |
| **CAD** | Q2 | ≤ 60 | 0.96 (0.87, 1.07) | 0.5 |  | 0.95 (0.86, 1.05) | 0.3 |  |
| **CAD** | Q3 | ≤ 60 | 1.14 (1.02, 1.26) | 0.017 |  | 1.01 (0.91, 1.12) | 0.9 |  |
| **CAD** | Q4 | ≤ 60 | 1.31 (1.17, 1.47) | <0.001 |  | 1.11 (0.98, 1.25) | 0.10 |  |
| **HF** |  | Sex |  |  | 0.013 |  |  | 0.405 |
| **HF** | ePVS | F |  |  |  |  |  |  |
| **HF** | Q1 | F | ref | ref |  | ref | ref |  |
| **HF** | Q2 | F | 0.98 (0.87, 1.10) | 0.7 |  | 1.05 (0.94, 1.18) | 0.4 |  |
| **HF** | Q3 | F | 1.23 (1.10, 1.38) | <0.001 |  | 1.24 (1.11, 1.39) | <0.001 |  |
| **HF** | Q4 | F | 1.49 (1.32, 1.69) | <0.001 |  | 1.40 (1.23, 1.59) | <0.001 |  |
| **HF** | ePVS | M |  |  |  |  |  |  |
| **HF** | Q1 | M | ref | ref |  | ref | ref |  |
| **HF** | Q2 | M | 1.23 (1.10, 1.38) | <0.001 |  | 1.19 (1.06, 1.33) | 0.003 |  |
| **HF** | Q3 | M | 1.44 (1.28, 1.62) | <0.001 |  | 1.28 (1.13, 1.44) | <0.001 |  |
| **HF** | Q4 | M | 2.10 (1.86, 2.38) | <0.001 |  | 1.64 (1.44, 1.86) | <0.001 |  |
| **HF** |  | Age |  |  | 0.603 |  |  | 0.066 |
| **HF** | ePVS | > 60 |  |  |  |  |  |  |
| **HF** | Q1 | > 60 | ref | ref |  | ref | ref |  |
| **HF** | Q2 | > 60 | 1.15 (1.04, 1.27) | 0.005 |  | 1.19 (1.08, 1.31) | <0.001 |  |
| **HF** | Q3 | > 60 | 1.38 (1.25, 1.53) | <0.001 |  | 1.31 (1.18, 1.44) | <0.001 |  |
| **HF** | Q4 | > 60 | 1.74 (1.57, 1.94) | <0.001 |  | 1.53 (1.37, 1.71) | <0.001 |  |
| **HF** | ePVS | ≤ 60 |  |  |  |  |  |  |
| **HF** | Q1 | ≤ 60 | ref | ref |  | ref | ref |  |
| **HF** | Q2 | ≤ 60 | 1.04 (0.90, 1.20) | 0.6 |  | 0.95 (0.83, 1.10) | 0.5 |  |
| **HF** | Q3 | ≤ 60 | 1.27 (1.09, 1.47) | 0.002 |  | 1.15 (1.00, 1.33) | 0.053 |  |
| **HF** | Q4 | ≤ 60 | 1.80 (1.54, 2.10) | <0.001 |  | 1.41 (1.20, 1.66) | <0.001 |  |
| **Stroke** |  | Sex |  |  | 0.578 |  |  | 0.59 |
| **Stroke** | ePVS | F |  |  |  |  |  |  |
| **Stroke** | Q1 | F | ref | ref |  | ref | ref |  |
| **Stroke** | Q2 | F | 0.94 (0.82, 1.07) | 0.3 |  | 1.09 (0.96, 1.24) | 0.2 |  |
| **Stroke** | Q3 | F | 1.11 (0.98, 1.27) | 0.11 |  | 1.19 (1.05, 1.36) | 0.007 |  |
| **Stroke** | Q4 | F | 1.31 (1.14, 1.51) | <0.001 |  | 1.25 (1.08, 1.45) | 0.003 |  |
| **Stroke** | ePVS | M |  |  |  |  |  |  |
| **Stroke** | Q1 | M | ref | ref |  | ref | ref |  |
| **Stroke** | Q2 | M | 1.05 (0.93, 1.19) | 0.4 |  | 1.00 (0.88, 1.13) | >0.9 |  |
| **Stroke** | Q3 | M | 1.23 (1.08, 1.41) | 0.002 |  | 1.09 (0.96, 1.25) | 0.2 |  |
| **Stroke** | Q4 | M | 1.45 (1.25, 1.68) | <0.001 |  | 1.18 (1.02, 1.38) | 0.029 |  |
| **Stroke** |  | Age |  |  | 0.445 |  |  | 0.694 |
| **Stroke** | ePVS | > 60 |  |  |  |  |  |  |
| **Stroke** | Q1 | > 60 | ref | ref |  | ref | ref |  |
| **Stroke** | Q2 | > 60 | 1.02 (0.92, 1.14) | 0.7 |  | 1.05 (0.94, 1.17) | 0.4 |  |
| **Stroke** | Q3 | > 60 | 1.15 (1.03, 1.28) | 0.017 |  | 1.12 (1.00, 1.25) | 0.047 |  |
| **Stroke** | Q4 | > 60 | 1.31 (1.16, 1.48) | <0.001 |  | 1.14 (1.00, 1.30) | 0.047 |  |
| **Stroke** | ePVS | ≤ 60 |  |  |  |  |  |  |
| **Stroke** | Q1 | ≤ 60 | ref | ref |  | ref | ref |  |
| **Stroke** | Q2 | ≤ 60 | 0.93 (0.80, 1.08) | 0.3 |  | 0.98 (0.84, 1.14) | 0.8 |  |
| **Stroke** | Q3 | ≤ 60 | 1.20 (1.03, 1.41) | 0.023 |  | 1.13 (0.97, 1.32) | 0.11 |  |
| **Stroke** | Q4 | ≤ 60 | 1.49 (1.25, 1.77) | <0.001 |  | 1.29 (1.08, 1.55) | 0.004 |  |

**Table S12.** Estimated cut-off points and 95% confidence intervals for admission and discharge ePVS across different endpoints.

| **Endpoints** | **ePVS_admission (95% CI)** | **ePVS_discharge (95% CI)** |
| --- | --- | --- |
| **CKM4** | 5.142 (5.023, 5.179) | 5.782 (5.637, 5.852) |
| **CKM4a** | 5.151 (5.132, 5.178) | 5.785 (5.574, 5.858) |
| **CKM4b** | 5.141 (5.082, 5.168) | 5.821(5.754, 5.945) |
| **CAD** | 5.148 (5.128, 5.185) | 5.775 (5.538, 5.938) |
| **HF** | 5.145 (5.083, 5.186) | 5.853 (5.787, 5.861) |
| **Stroke** | 5.136 (5.002, 5.193) | 5.776 (5.536, 5.924) |

# Table S13. Associations between ePVS quartiles and clinical outcomes in the parsimonious model

|  |  | **ePVS_admission** | | **ePVS_discharge** | |
| --- | --- | --- | --- | --- | --- |
| **Endpoints** | **Characteristic** | **HR (95% CI)** | ***P* value** | **HR (95% CI)** | ***P* value** |
| **CKM4** | Q1 | 1.00 (reference) | ref | 1.00 (reference) | ref |
|  | Q2 | 1.04 (0.99, 1.08) | 0.091 | 1.10 (1.06, 1.15) | <0.001 |
|  | Q3 | 1.08 (1.05, 1.12) | <0.001 | 1.16 (1.12, 1.20) | <0.001 |
|  | Q4 | 1.36 (1.25, 1.48) | <0.001 | 1.35 (1.24, 1.47) | <0.001 |
| **CKM4a** | Q1 | 1.00 (reference) | ref | 1.00 (reference) | ref |
|  | Q2 | 1.07 (0.95, 1.22) | 0.275 | 1.16 (1.02, 1.32) | 0.022 |
|  | Q3 | 1.13 (1.08, 1.18) | <0.001 | 1.20 (1.15, 1.26) | <0.001 |
|  | Q4 | 1.33 (1.16, 1.53) | <0.001 | 1.38 (1.20, 1.59) | <0.001 |
| **CKM4b** | Q1 | 1.00 (reference) | ref | 1.00 (reference) | ref |
|  | Q2 | 1.07 (1.03, 1.11) | <0.001 | 1.10 (1.06, 1.14) | <0.001 |
|  | Q3 | 1.83 (1.60, 2.09) | <0.001 | 1.83 (1.60, 2.10) | <0.001 |
|  | Q4 | 1.86 (1.59, 2.18) | <0.001 | 1.88 (1.61, 2.21) | <0.001 |
| **CAD** | Q1 | 1.00 (reference) | ref | 1.00 (reference) | ref |
|  | Q2 | 1.02 (0.98, 1.06) | 0.339 | 1.00 (0.96, 1.04) | 0.950 |
|  | Q3 | 1.16 (1.12, 1.20) | <0.001 | 1.25 (1.21, 1.30) | <0.001 |
|  | Q4 | 1.40 (1.22, 1.59) | <0.001 | 1.45 (1.27, 1.66) | <0.001 |
| **HF** | Q1 | 1.00 (reference) | ref | 1.00 (reference) | ref |
|  | Q2 | 1.20 (1.10, 1.32) | <0.001 | 1.18 (1.08, 1.29) | <0.001 |
|  | Q3 | 1.43 (1.32, 1.55) | <0.001 | 1.42 (1.31, 1.54) | <0.001 |
|  | Q4 | 1.52 (1.23, 1.89) | <0.001 | 1.54 (1.24, 1.91) | <0.001 |
| **Stroke** | Q1 | 1.00 (reference) | ref | 1.00 (reference) | ref |
|  | Q2 | 0.95 (0.76, 1.18) | 0.648 | 1.15 (1.09, 1.20) | <0.001 |
|  | Q3 | 1.15 (1.10, 1.21) | <0.001 | 1.13 (1.07, 1.19) | <0.001 |
|  | Q4 | 1.28 (1.13, 1.44) | <0.001 | 1.45 (1.28, 1.64) | <0.001 |

The parsimonious model was adjusted for age, sex, eGFR, diabetes mellitus, hypertension, and ACEI. Quartile 1 (Q1) serves as the reference category.

# Table S14. Fine-Gray competing risk models for progression to CKM stage 4b

| **Competing event** | **Exposure** | **sHR (95% CI)** | ***P* value** |
| --- | --- | --- | --- |
| **All-cause mortality** | ePVS_admission | 1.18 (1.15–1.20) | <0.001 |
| **All-cause mortality** | ePVS_discharge | 1.31 (1.27–1.35) | <0.001 |
| **CKM 4a** | ePVS_admission | 1.08 (1.06–1.10) | <0.001 |
| **CKM 4a** | ePVS_discharge | 1.12 (1.09–1.14) | <0.001 |

#Adjusted with sex, age, LOS, DM, hypertension, hypertriglyceridemia, MetS, CKD, anemia, hyperuricemia; bicarbonate, chloride, eGFR, platelet count, potassium, sodium, WBC, metformin, glimepiride, furosemide, beta-block, statin, CCB, ARB, and ACEI.

# Figure S1. The forest plot for ePVS_discharge in five models. (Model 0: unadjusted; Model 1: adjusted with sex, age, and LOS; Model 2: adjusted with Model 1 and DM, hypertension, hypertriglyceridemia, MetS, CKD, anemia, hyperuricemia; Model 3: adjusted with Model 2 and bicarbonate, chloride, eGFR, platelet count, potassium, sodium, and WBC; Model 4: adjusted with Model 3 and metformin, glimepiride, furosemide, beta-block, statin, CCB, ARB, and ACEI)

# Figure S2. The RCS analysis for various endpoints with the ePVS_discharge

# Figure S3. The Cumulative Risk Curve for various endpoints with ePVS_discharge quartiles

# Figure S4. The Cumulative Risk Curve for various endpoints with ePVS_admission > 5.14 or not

# Figure S5. The Cumulative Risk Curve for various endpoints with ePVS_discharge >5.78 or not
